# The Application of Trauma-Informed Care to Health Care for Military-Connected Individuals

**DOI:** 10.15766/mep_2374-8265.11466

**Published:** 2024-11-05

**Authors:** Binny Chokshi, Meaghan Wido, Sarah Prabhakar, Elizabeth Hisle-Gorman

**Affiliations:** 1 Associate Professor, Division of Military Child and Family Research, Department of Pediatrics, Uniformed Services University of the Health Sciences F. Edward Hébert School of Medicine; 2 Assistant Professor, Department of Pediatrics, Uniformed Services University of the Health Sciences F. Edward Hébert School of Medicine; 3 Data Analyst, Henry M. Jackson Foundation for the Advancement of Military Medicine employee under contract with the Department of Pediatrics, Uniformed Services University of the Health Sciences; 4 Associate Professor, Department of Pediatrics, and Director, Division of Military Child and Family Research, Uniformed Services University of the Health Sciences F. Edward Hébert School of Medicine

**Keywords:** Military Health Care, Toxic Stress, Trauma-Informed Care, Case-Based Learning, Curriculum Development, Health Equity, Pediatrics, Population Health, Primary Care

## Abstract

**Introduction:**

Military families face unique stressors beyond civilian life, such as deployments, frequent relocations, and the potential for combat, all of which can significantly impact well-being. A trauma-informed care (TIC) approach to military medicine is paramount; however, a critical gap exists, with no published curricula to guide practitioners in employing TIC in the care of military-connected individuals.

**Methods:**

We delivered a 50-minute interactive and virtual session to second-year medical students at the Uniformed Services University (USU) that reviewed the neurobiology of adversity and the relevance of TIC in caring for military-connected populations. Participants completed a 14-question pre- and posttest on perceived knowledge, attitudes, practice, and confidence, as well as posttest questions evaluating session quality. The USU Institutional Review Board approved this evaluation.

**Results:**

One hundred sixty medical students participated in the session, with 78 matched pre- and posttest responses. We observed a statistically significant pre-post improvement (*p* ≤ .05) in all category scores, with the largest changes in knowledge (1.33) and confidence (1.33). On a 5-point Likert scale, with 5 being best, mean scores for overall quality of the session and relevance of the material to participants’ learning and future practice were 3.95 and 4.20, respectively.

**Discussion:**

By equipping health care providers with knowledge and confidence to apply TIC in military medicine, we can improve the well-being of service members and their families across both military and civilian health care settings. Broader implementation of this program has potential to improve patient outcomes and overall health care delivery for this population.

## Educational Objectives

By the end of this activity, learners will be able to:

1. Define trauma and toxic stress and their connection to health.
2. Describe the importance and components of a trauma-informed approach to care.
3. Identify the relevance of trauma-informed care to military medicine.

## Introduction

Nearly 90% of US adults in a national sample reported traumatic event exposure, and exposure to multiple trauma types was common prior to age 18.^1^ The Substance Abuse and Mental Health Services Administration defines trauma as an event, series of events, or set of circumstances that is experienced by an individual as physically or emotionally harmful or life threatening and that has lasting adverse effects on the individual's functioning and mental, physical, social, emotional, or spiritual well-being.^2^

The landmark adverse childhood experiences (ACEs) study demonstrated the powerful impact of childhood traumatic exposures on physical and mental health outcomes in adulthood.^3^ Subsequent work has shown that any experience, at any age, that activates a stress response that is prolonged, severe, or chronic can be considered traumatic and have health impacts over the life course.^4^

A consideration of trauma exposure is particularly important when considering the 2.6 million uniformed US service members and their families, as military life in the US is inherently associated with experiencing potentially stressful or traumatic events, such as combat deployments, family separations, and a high-demand workplace. Military dependents (i.e., spouses/partners, children) also experience prolonged parental separation related to deployment and trainings, multiple relocations, and injury or death of the service member. While some of these factors may lead to positive effects, such as developing coping skills, adaptability, and autonomy, these military-specific situations can also adversely impact service members and their loved ones. Furthermore, US male service members are more likely to self-report higher rates of premilitary ACEs than their nonmilitary male peers. Those with higher premilitary ACE exposure may be drawn to volunteer for service, seeking to escape an adverse home life and attracted by the promise of a pay and benefits.^5–7^ This is noteworthy, as service members with a history of ACEs have higher rates of mental health challenges when exposed to military-specific stressors, such as deployment-related traumas, than service members with no ACE history, and understanding service member ACE profiles could help to identify those who may be in particular need of mental health support and resources in the face of military-specific challenges.^6,7^

The impact of cumulative adversity is reflected in the health outcomes of US veterans, which show that veterans, compared to those who not served, have a significantly higher risk of obesity and related comorbidities and are also more likely to suffer from mental health disease, including suicide.^8^ A study by Eaton and colleagues underscores the potential impact of deployments on military families: They found that 16.9% of military spouses seeking primary care during their partner's deployment reported moderate to severe emotional problems, alcohol use, or family issues.^9^ Military-connected youth have also been shown to have increased mental health concerns in response to parental deployments, injuries, and multiple relocations.^10–12^ These statistics highlight the necessity for a trauma-informed approach to support the overall health and wellness of military-connected individuals—a necessity in both civilian and military health care facilities, as 60% of military health care is provided in civilian settings.^13^

Trauma-informed care (TIC) is a strengths-based approach to health care that acknowledges the widespread exposure to and health impact of traumatic events. TIC seeks to promote psychological and physical safety within the health care setting to allow for patient-provider collaborations rooted in trust and mutual understanding. According to the Substance Abuse and Mental Health Services Administration, a trauma-informed approach to health care requires a recognition of the physical and mental health manifestations of trauma, with a response that fully integrates knowledge about trauma into policies, procedures, and practices.^2^ The use of TIC has been shown to improve health outcomes, doctor-patient relationships, and patient satisfaction.^14–18^

Despite its applicability, there is a lack of curricula or guidance on the inclusion of TIC within military populations. *MedEdPORTAL* has published curricular resources related to ACEs and TIC.^19–24^ While these resources provide detailed background information on the science of adversity and the principles of TIC, they lack a focus on military-connected individuals and the specific stressors they can be exposed to, such as deployments, relocations, and parental injury, as well as how these experiences can lead to a toxic stress response with resultant impact on health. While *MedEdPORTAL* also has published curricular resources centering on the unique factors and stressors associated with military life, their content emphasizes the care of veterans and does not include a focus on those currently serving and their families. These also lack a focus on translation to clinical practice and a discussion of TIC.^25–27^

As a result, we set out to create an educational session on the critical need for TIC and its relevance to military medicine for medical students at the Uniformed Services University of the Health Sciences (USU). Following Kern's model of curricular development,^28^ the problem was identified as delineated above. Discussion with pediatrics, family medicine, and psychiatry clerkship directors guided a targeted needs assessment, highlighting the need for formalized instruction on TIC in the preclinical years for USU medical students. Finally, following successful presentation and discussion with an education council committee, our TIC program gained approval as a required session for all second-year medical students at USU before they initiated their clinical clerkships.

## Methods

### Curricular Context

We delivered this 50-minute, interactive session (Appendix A) virtually to 160 second-year medical students at USU. Learners did not have to have any prerequisite knowledge but were provided with optional prereading requiring 15–20 minutes of learner time. This included a link to information on the original ACEs study on the Centers for Disease Control website, a 3-minute video explaining TIC from the Center for Health Care Strategies’ Trauma-Informed Care Implementation Resource Center, and a two-page information sheet from the Psychological Health Center of Excellence on TIC and military populations.^29–31^ A message about the session, the Educational Objectives, and the prereading was sent to all learners in the week prior to the session (Appendix B). This message also included a link to the pretest (Appendix C). The didactic portion of the session was facilitated by a faculty member (Binny Chokshi) with prior knowledge of TIC and experience in development and delivery of TIC-related health care content. The breakout rooms were facilitated by nine faculty members at USU, all of whom had some basic knowledge of the impact of adversity on health and the principles of TIC. A facilitator guide (Appendix D) was emailed to all facilitators the day before the session.

### Resource Content

After identifying the problem and needs assessment as highlighted above, we developed the Educational Objectives for this session through a literature review and assessment of published curricula on the application of TIC in health care. The educational strategies were intentionally created to include active learning, audience response, small-group discussion, and group reflection. The first 30 minutes of the session featured information on the relationship between adversity, toxic stress, and poor health and behavioral outcomes, followed by a review of the literature related to ACE exposure in service members. The slides reviewed how military-specific stressors, such as deployments, relocations, injuries, and combat exposure, could be considered potentially traumatic, as they could lead to a toxic stress response and resultantly have a health impact on service members, their partners, and their children. To increase the active learning during this module, we utilized audience response polling (PollEverywhere) for short-answer and word-cloud questions. Participants then received detailed information on TIC. This included information about the central tenet of TIC (shifting the focus from “What's wrong with you?” to “What happened to you?”)^32^ and principles of TIC. Next, participants were divided randomly into virtual breakout rooms with faculty facilitators. Within small groups, students explored how an understanding of TIC could influence their approach to patient care in the coming months, while also brainstorming ways to integrate the science of trauma and its impact on health into military medical settings. Participants collectively discussed a case related to a young service member who was expecting a child and wanted to quit smoking. The case intentionally highlighted diagnoses and behaviors (adolescent parenthood, smoking) that could be linked to a history of traumatic exposures.

### Evaluation

We developed the evaluation based on similar evaluator mechanisms published in *MedEdPORTAL* aiming to evaluate the impact of a onetime curricular session.^21,22^ Participants completed a pretest with 14 questions, scored on 5-point Likert scales, in categories of perceived knowledge (six questions), practice (four questions), attitudes (three questions), and confidence (one question). A posttest at the end of the session was linked by QR code and included the same 14 questions from the pretest, along with four additional questions related to the quality of the session. Participants were asked to include a unique identifier in order to match individual pre- and posttest responses. This evaluation was approved by the USU Institutional Review Board.

We utilized descriptive statistics to highlight means and standard deviations across the pre- and posttest surveys. We used a paired *t* test to determine statistically significant differences through the generation of *p* values and 95% confidence intervals for mean differences in scores between pre- and postsession responses. We calculated a knowledge, attitudes, and practice overall score by taking the mean of the items included in each domain. We applied descriptive content analysis to the final question of the posttest, asking participants to list one specific way they would apply the session to their clerkship practice.

## Results

A total of 160 second-year medical students at USU participated in the virtual session. Overall, 156 participants completed the pretest (response rate: 97%), and 108 completed the posttest (response rate: 68%), with 78 paired responses.

We observed a statistically significant improvement (*p* ≤ .05) in all category scores for the posttest compared with the pretest (Table 1). Each individual item in the knowledge and confidence categories had a statistically significant increase in pre- to posttest mean score. The categories of attitudes and practice demonstrated high pretest mean scores for each item (>4.0). On a 5-point Likert scale with 5 being the best, the mean score for the overall quality of the session was 4.0, and for relevance of the material to participants’ learning and future practice, as well as for clarity and appropriateness of the session to participants’ level of training, mean scores were 4.2.

**Table 1.**
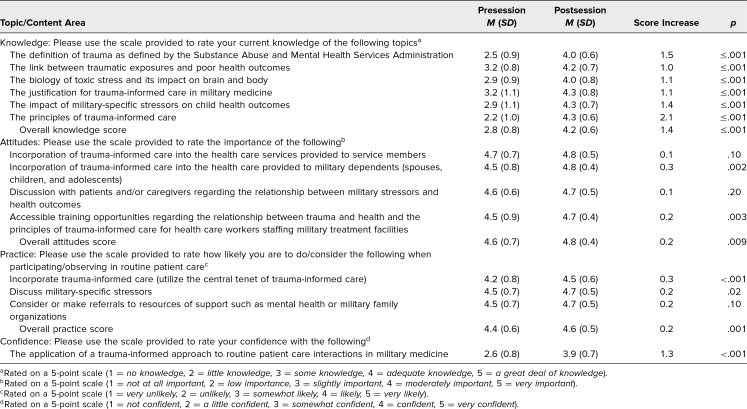
Mean Scores and Score Differences in Paired Pre- and Postsession Surveys (*N* = 79)

For the final question of the postsurvey, descriptive content analysis revealed five prominent categories of responses, as shown in Table 2. Participants most commonly remarked that they would apply the session during their clerkship practice by being mindful of the link between trauma and patient behaviors and trauma and health outcomes. Participants also remarked on their desire to provide nonjudgmental care, collaborate with patients, and utilize the central tenet of TIC. Lastly, participants mentioned that in their future rotations, they would consider the reality of military-specific stressors and their potential impact on health and wellness.

**Table 2.**
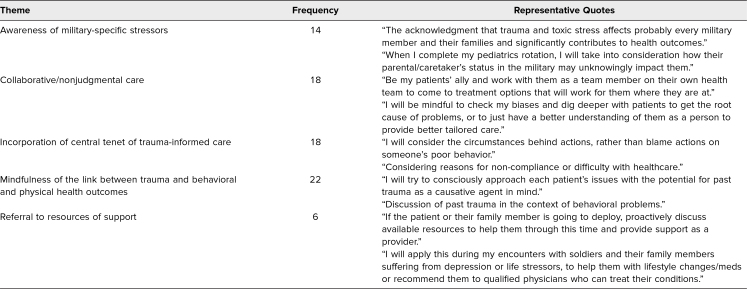
Specific Ways Participants Will Apply the Session to Their Clerkship Practice

## Discussion

This curriculum addressed an important educational gap for undergraduate medical students, providing a concise introductory session on the application of a trauma-informed approach to health care delivery for military-connected individuals. Prior to the session, participants believed in the importance of considering and utilizing a trauma-informed approach in military populations, but they demonstrated a lack of perceived knowledge and confidence in TIC provision. Our evaluation demonstrated that the 50-minute, interactive session was successful in increasing participants’ perceived knowledge, confidence, attitudes, and practice regarding the application of TIC to the health care provided to military-connected individuals. Lack of statistical significance for individual items in the attitudes and practice categories can be explained by the high pretest mean scores for each item (>4.0). Participants also found this session to be relevant to their learning and practice and appropriate to their level of training.

Notably, due to the barrier of time, this session does not discuss the immense benefits associated with military life and culture, such as access to universal health care, secure salary, childcare, and numerous sources of support. These can contribute to positive experiences for military-connected individuals and can mitigate the impact of adversity. For sites aiming to implement this session, we suggest inclusion of information on positive childhood experiences and resources of support, such as Military OneSource or local military family life counselor contact information.^33^ These can be embedded within additional patient cases as appropriate to facilitate active application of content by brainstorming resource referrals. At our institution, we have addressed this barrier by implementing a 1-hour required reflective practice session during the third-year pediatrics clerkship that introduces the concept and science of positive childhood experiences. Students are invited to reflect on how to balance a trauma-informed approach to military health care with the promotion of positive childhood experiences. In addition, we are exploring the option to partner with our interdisciplinary colleagues to further provide information on trauma-informed interventions for service members and their dependents during additional clerkship rotations.

Another limitation of our session and evaluation is single-site implementation, though this curriculum could be easily adapted in additional settings. We plan to partner with educational faculty at larger military treatment facilities that host USU medical students for clerkships and have graduate medical education training programs to foster broader implementation of this session. There are over 700 military treatment facilities within the US and globally that serve US service members, retirees, and their families, and 60% of military health care is provided in civilian settings, highlighting the broad applicability and potential utility of this information.^13,34^ It can also be delivered to additional audiences, such as residents, attending physicians, and interdisciplinary health care professionals, including nurses and physician assistants. In addition, while our session has been delivered virtually, it can be easily adapted to an in-person environment. For those looking to implement this session at their institutions, we suggest obtaining buy-in from educational leadership, which helped us to deliver the session to all second-year medical students during a week of required coursework. We also encourage discussion with educational leaders or participants themselves to ensure content is adapted for relevance, for instance, that patient cases are applicable to the patient population of the specific military setting (branch, location).

Lastly, a limitation of our evaluation is the immediate posttesting, which does not allow for an evaluation of knowledge retention and application in practice. Future evaluative efforts may include a qualitative analysis of participant reflections in the 3- to 6-month period after completing the session to understand its utility and relevance to clinical care during the clerkship year. Potential additional opportunities for both instruction and evaluation include utilizing the simulation center at USU to allow students to directly practice their trauma-informed communication and physical examination skills while working through relevant patient cases.

## Appendices


Slide Set.pptxPresession Message.docxPre-Post Evaluation.docxFacilitator Guide.docx
*All appendices are peer reviewed as integral parts of the Original Publication.*

## References

[1] Kilpatrick DG, Resnick HS, Milanak ME, Miller MW, Keyes KM, Friedman MJ. National estimates of exposure to traumatic events and PTSD prevalence using *DSM-IV* and *DSM-5* criteria. J Trauma Stress. 2013;26(5):537–547. 10.1002/jts.21848 24151000 PMC4096796

[2] Substance Abuse and Mental Health Services Administration. SAMHSA's Concept of Trauma and Guidance for a Trauma-Informed Approach. Substance Abuse and Mental Health Services Administration; 2014. HHS publication SMA 14-4884.

[3] Felitti VJ, Anda RF, Nordenberg D, et al. Relationship of childhood abuse and household dysfunction to many of the leading causes of death in adults: the Adverse Childhood Experiences (ACE) Study. Am J Prev Med. 1998;14(4):245–258. 10.1016/S0749-3797(98)00017-8 9635069

[4] Shonkoff JP, Garner AS; Committee on Psychosocial Aspects of Child and Family Health; Committee on Early Childhood, Adoption, and Dependent Care; Section on Developmental and Behavioral Pediatrics. The lifelong effects of early childhood adversity and toxic stress. Pediatrics. 2012;129(1):e232–e246. 10.1542/peds.2011-2663 22201156

[5] Blosnich JR, Dichter ME, Cerulli C, Batten SV, Bossarte RM. Disparities in adverse childhood experiences among individuals with a history of military service. JAMA Psychiatry. 2014;71(9):1041–1048. 10.1001/jamapsychiatry.2014.724 25054690 PMC8981217

[6] Sareen J, Henriksen CA, Bolton SL, Afifi TO, Stein MB, Asmundson GJG. Adverse childhood experiences in relation to mood and anxiety disorders in a population-based sample of active military personnel. Psychol Med. 2013;43(1):73–84. 10.1017/S003329171200102X 22608015

[7] Reed-Fitzke K, Duncan JM, Wojciak AS, Ferraro AJ, Sánchez J, Smith KM. A person-centered approach to identifying at-risk U.S. army soldiers-in-training based on adverse childhood experiences. Traumatology. 2023;29(4):481–492. 10.1037/trm0000395

[8] Betancourt JA, Granados PS, Pacheco GJ, et al. Exploring health outcomes for U.S. veterans compared to non-veterans from 2003 to 2019. Healthcare (Basel). 2021;9(5):604. 10.3390/healthcare9050604 34070037 PMC8158130

[9] Eaton KM, Hoge CW, Messer SC, et al. Prevalence of mental health problems, treatment need, and barriers to care among primary care-seeking spouses of military service members involved in Iraq and Afghanistan deployments. Mil Med. 2008;173(11):1051–1056. 10.7205/MILMED.173.11.1051 19055177

[10] Gorman GH, Eide M, Hisle-Gorman E. Wartime military deployment and increased pediatric mental and behavioral health complaints. Pediatrics. 2010;126(6):1058–1066. 10.1542/peds.2009-2856 21059715

[11] Cunitz K, Dölitzsch C, Kösters M, et al. Parental military deployment as risk factor for children's mental health: a meta-analytical review. Child Adolesc Psychiatry Ment Health. 2019;13:26. 10.1186/s13034-019-0287-y 31249614 PMC6587296

[12] Hisle-Gorman E, Susi A, Gorman GH. The impact of military parents’ injuries on the health and well-being of their children. Health Aff (Millwood). 2019;38(8):1358–1365. 10.1377/hlthaff.2019.00276 31381386

[13] Approaches to Changing Military Health Care. Congressional Budget Office; 2017. Accessed October 11, 2024. https://www.cbo.gov/system/files/115th-congress-2017-2018/reports/53137-approachestochangingmilitaryhealthcare.pdf

[14] Stillerman A, Altman L, Peña G, et al. Advancing trauma-informed care in hospitals: the time is now. Perm J. 2023;27(1):16–20. 10.7812/TPP/22.081 36428252 PMC10013720

[15] Agboola IK, Coupet E Jr, Wong AH. “The coats that we can take off and the ones we can't”: the role of trauma-informed care on race and bias during agitation in the emergency department. Ann Emerg Med. 2021;77(5):493–498. 10.1016/j.annemergmed.2020.11.021 33579587 PMC8085054

[16] Purkey E, Davison C, MacKenzie M, et al. Experience of emergency department use among persons with a history of adverse childhood experiences. BMC Health Serv Res. 2020;20:455. 10.1186/s12913-020-05291-6 32448175 PMC7245948

[17] Tomaz T, Castro-Vale I. Trauma-informed care in primary health settings—which is even more needed in times of COVID-19. Healthcare (Basel). 2020;8(3):340. 10.3390/healthcare8030340 32937966 PMC7551418

[18] Noroña-Zhou AN, Ashby BD, Richardson G, et al. Rates of preterm birth and low birth weight in an adolescent obstetric clinic: achieving health equity through trauma-informed care. Health Equity. 2023;7(1):562–569. 10.1089/heq.2023.0075 37731783 PMC10507928

[19] Chokshi B, Walsh K, Dooley D, Falusi O, Deyton L, Beers L. Teaching trauma-informed care: a symposium for medical students. MedEdPORTAL. 2020;16:11061. 10.15766/mep_2374-8265.11061 33409358 PMC7780743

[20] Schmitz A, Light S, Barry C, Hodges K. Adverse childhood experiences and trauma-informed care: an online module for pediatricians. MedEdPORTAL. 2019;15:10851. 10.15766/mep_2374-8265.10851 31934614 PMC6952282

[21] Pletcher BA, O'Connor M, Swift-Taylor ME, DallaPiazza M. Adverse childhood experiences: a case-based workshop introducing medical students to trauma-informed care. MedEdPORTAL. 2019;15:10803. 10.15766/mep_2374-8265.10803 30931382 PMC6415008

[22] Chokshi B, Chen KLD, Beers L. Interactive case-based childhood adversity and trauma-informed care electronic modules for pediatric primary care. MedEdPORTAL. 2020;16:10990. 10.15766/mep_2374-8265.10990 33094156 PMC7549390

[23] Brennan EF, Markopoulos A, Rodriguez J, Sheth NK, Shah N. Addressing a gap in medical school training: identifying and caring for human trafficking survivors using trauma-informed care. MedEdPORTAL. 2023;19:11304. 10.15766/mep_2374-8265.11304 36926052 PMC10011204

[24] Lee CH, Dos Santos C, Brown T, Ashworth H, Lewis JJ. Trauma-informed care for acute care settings: a novel simulation training for medical students. MedEdPORTAL. 2023;19:11327. 10.15766/mep_2374-8265.11327 37520013 PMC10376910

[25] Goldenberg M, Hamaoka DA, Santiago PN, McCarroll JE. Basic training: a primer on military life and culture for health care providers and trainees. MedEdPORTAL. 2012;8:9270. 10.15766/mep_2374-8265.9270

[26] Fabrizio J, DeNardi K, Boland M, Suffoletto JA. Use of a standardized patient in teaching medical students to assess for PTSD in military veteran patients. MedEdPORTAL. 2017;13:10608. 10.15766/mep_2374-8265.10608 30800810 PMC6338190

[27] Day H, Scott R, Fulmer V, et al. Women's health issues in military veterans: standardized patient cases in motivational interviewing, a case materials guide. MedEdPORTAL. 2013;9:9539. 10.15766/mep_2374-8265.9539

[28] Thomas PA, Kern DE, Hughes MT, Chen BY, eds. Curriculum Development for Medical Education: A Six-Step Approach. 3rd ed. Johns Hopkins University Press; 2016.

[29] About the CDC-Kaiser ACE study. Centers for Disease Control and Prevention. Updated April 6, 2021. Accessed October 11, 2024. https://www.cdc.gov/violenceprevention/aces/about.html

[30] Center for Health Care Strategies. What Is Trauma-Informed Care? YouTube. January 23, 2019. Accessed October 11, 2024. https://youtu.be/fWken5DsJcw

[31] Health Care Provider's Guide to Trauma-Informed Care. Psychological Health Center of Excellence; 2018. Publication PUID 4724. Accessed October 11, 2024. https://jko.jten.mil/courses/CTIP_healthcare_toolkit/courseFiles/ContentPages/CoursePages/resources/PHCoE_TraumaProviderBrochure_v0.9_Final%20508_07MAR2018_.pdf

[32] What is trauma-informed care? Trauma-Informed Care Implementation Resource Center. Accessed October 11, 2024. https://www.traumainformedcare.chcs.org/what-is-trauma-informed-care/

[33] Non-medical counseling. Military OneSource. Accessed October 11, 2024. https://www.militaryonesource.mil/non-medical-counseling

[34] Defense Health Care: DOD Should Reevaluate Market Structure for Military Medical Treatment Facility Management. US Government Accountability Office; 2023. Publication GAO-23-105441. Accessed October 11, 2024. https://www.gao.gov/assets/d23105441.pdf

