# Supplementary material for: The Application of Trauma-Informed Care to Health Care for Military-Connected Individuals
Source: MedEdPORTAL. 2024 Nov 5;20:11466. doi: 10.15766/mep_2374-8265.11466 (PMC11534622; doi:10.15766/mep_2374-8265.11466)
Supplement: Supplementary file 1 — Slide Set.pptxPresession Message.docxPre-Post Evaluation.docxFacilitator Guide.docx [file mep_2374-8265.11466-s001.zip › C. Pre-Post Evaluation.docx]

**Appendix C: Pretest and Posttest Evaluation**

**Instructions:** This is the pre/postsurvey for students to complete prior to or at the beginning of The Application of Trauma-Informed Care to Healthcare for Military Connected Individuals session. We recommend loading the pre and post survey into an online survey tool and including a QR code/link to the survey in the PowerPoint to facilitate learner access. As the scale for each set of questions is listed before the questions, if distributing this via paper, you will need to reformat the file to add response options and print enough copies prior to the workshop.

**PRETEST**

Please use the following to create a unique identifier that will allow us to track survey data anonymously:

1. Last letter of your first name
2. Last two digits of your phone number
3. First two letters of your elementary school

Example: Mary Smith 202 476 5075, Village Elementary = Y75VI

Knowledge:

1. No knowledge
2. Little knowledge
3. Some knowledge
4. Adequate knowledge
5. A great deal of knowledge

**Please use the scale above to rate your current knowledge on the following topics:**

1. The definition of trauma as defined by the Substance Abuse and Mental Health Services Administration
2. The link between traumatic exposures and poor health outcomes
3. The biology of toxic stress and its impact on the brain and body
4. The justification for trauma-informed care in military medicine
5. The impact of military-specific stressors on child health outcomes
6. The central tenet of trauma-informed care

Attitudes:

1. Not at all important
2. Low importance
3. Slightly important
4. Moderately important
5. Very important

**Please use the scale above to rate the importance of the following:**

1. Incorporation of trauma-informed care into the healthcare services provided to service members
2. Incorporation of trauma-informed care into the health services provided to military dependents (spouses, children, and adolescents)
3. Discussion with patients and/or caregivers regarding the relationship between military-specific stressors and health outcomes
4. Discussion with patients and/or caregivers regarding promotion of positive childhood experiences
5. Accessible training opportunities regarding the relationship between trauma and health and the principles of trauma-informed care for healthcare workers staffing military treatment facilities

Practice:

1. Very unlikely
2. Unlikely
3. Somewhat likely
4. Likely
5. Very likely

**Please use the scale above to indicate how likely you are to do or consider the following when observing/participating in routine patient care:**

1. Incorporate trauma-informed care (utilize the central tenet of trauma-informed care)
2. Discuss military-specific stressors
3. Consider making referrals to resources of support such as mental health or military family organizations

Confidence**:**

1. Not confident
2. Little confidence
3. Somewhat confident
4. Confident
5. Very confident

**Please use the scale above to rate your confidence with**

1. The application of a trauma-informed approach to routine patient care interactions in military medicine

**POSTTEST**

Please use the following to create a unique identifier that will allow us to track survey data anonymously:

1. Last letter of your first name
2. Last two digits of your phone number
3. First two letters of your elementary school

Example: Mary Smith 202 476 5075, Village Elementary = Y75VI

Knowledge:

1. No knowledge
2. Little knowledge
3. Some knowledge
4. Adequate knowledge
5. A great deal of knowledge

**Please use the scale above to rate your current knowledge on the following topics:**

1. The definition of trauma as defined by the Substance Abuse and Mental Health Services Administration
2. The link between traumatic exposures and poor health outcomes
3. The biology of toxic stress and its impact on the brain and body
4. The justification for trauma-informed care in military medicine
5. The impact of military-specific stressors on child health outcomes
6. The central tenet of trauma-informed care

Attitudes:

1. Not at all important
2. Low importance
3. Slightly important
4. Moderately important
5. Very important

**Please use the scale above to rate the importance of the following:**

1. Incorporation of trauma-informed care into the healthcare services provided to service members
2. Incorporation of trauma-informed care into the health services provided to military dependents (spouses, children, and adolescents)
3. Discussion with patients and/or caregivers regarding the relationship between military-specific stressors and health outcomes
4. Discussion with patients and/or caregivers regarding promotion of positive childhood experiences
5. Accessible training opportunities regarding the relationship between trauma and health and the principles of trauma-informed care for healthcare workers staffing military treatment facilities

Practice:

1. Very unlikely
2. Unlikely
3. Somewhat likely
4. Likely
5. Very likely

**Please use the scale above to indicate how likely you are to do or consider the following when observing/participating in routine patient care:**

1. Incorporate trauma-informed care (utilize the central tenet of trauma-informed care)
2. Discuss military-specific stressors
3. Consider making referrals to resources of support such as mental health or military family organizations

Confidence**:**

1. Not confident
2. Little confidence
3. Somewhat confident
4. Confident
5. Very confident

**Please use the scale above to rate your confidence with:**

- - - 1. The application of a trauma-informed approach to routine patient care interactions in military medicine

Additional Questions:

1. Please rate the quality of the module:

1. Very poor
2. Poor
3. Fair
4. Good
5. Very good

2. This session was relevant to my learning and future clinical practice:

1. Strongly disagree
2. Disagree
3. Neutral
4. Agree
5. Strongly agree

3. The content of this module was clear and appropriate to my level of training:

1. Strongly disagree
2. Disagree
3. Neutral
4. Agree
5. Strongly agree
6. Please list one specific way you will apply this session to your clerkship practice.
