# Supplementary material for: The Application of Trauma-Informed Care to Health Care for Military-Connected Individuals
Source: MedEdPORTAL. 2024 Nov 5;20:11466. doi: 10.15766/mep_2374-8265.11466 (PMC11534622; doi:10.15766/mep_2374-8265.11466)
Supplement: Supplementary file 1 — Slide Set.pptxPresession Message.docxPre-Post Evaluation.docxFacilitator Guide.docx [file mep_2374-8265.11466-s001.zip › D. Facilitator Guide.docx]

**Appendix D: Facilitator Guide**

**Transition to Clerkship: A Trauma-Informed Approach to Military Medicine**

**Instructions:** This facilitator guide is intended to help prepare breakout room/small group facilitators of the Trauma-Informed Approach to Military Medicine session. All facilitators should review this guide in full at least 1 day in advance of the session to ensure that they are prepared to implement the workshop effectively.

Time: 50 minutes

- 5 minutes: Introductions (pretest)
- 10 minutes: Slides 2-20, review of ACEs study and PollEverywhere questions
- 10 minutes: Importance to military medicine
- 5 minutes: Review of TIC
- 15 minutes: Breakout rooms
- 5 minutes: Closing (post-test)

*Optional* Prereading/Viewing for Facilitator Prep:

- About the CDC-Kaiser ACE Study^1^
- <https://www.cdc.gov/violenceprevention/aces/about.html>
- *Informational website with summarized content regarding the the original ACES study, with linked information about the study questionnaires, data and statistics, and major findings.*
- [What](https://www.youtube.com/watch?v=fWken5DsJcw) is Trauma-Informed Care?^2^
- <https://youtu.be/fWken5DsJcw>
- *Informational video from the Center for Health Care Strategies, Trauma-Informed Care Implementation Research Center.*
- [Health](https://www.qmo.amedd.army.mil/ptsd/PHCoE_TraumaProviderBrochure_v0.9_Final%20508_07MAR2018_.pdf) Care Provider’s Guide to Trauma-Informed Care^3^
- <https://jko.jten.mil/courses/CTIP_healthcare_toolkit/courseFiles/ContentPages/CoursePages/resources/PHCoE_TraumaProviderBrochure_v0.9_Final%20508_07MAR2018_.pdf>
- *A 2-page informational guide on the principles of trauma-informed care as they could apply to military service members and veterans, with a list of pertinent resources.*
- The Health and Readiness of Service Members: ACEs to PACEs^4^
- <https://pubmed.ncbi.nlm.nih.gov/32074355/>
- *Published article in Military Medicine related to adverse and positive childhood experiences of a small cohort of US service members.*

Breakout Room Facilitation: (Total Time: 15 minutes) Discussion Prompts and Suggested Probing Questions:

1. **How do you think this will change your approach to patient care in the next few months?**
   1. What disciplines within medicine is trauma-informed care applicable to?
   2. What are barriers / facilitators to including trauma-informed care in clinical medicine?

1. **How can the science of trauma and the impact on health be incorporated into military medicine?**
   1. Should there be screening for adversity in clinics? How could this be helpful?/harmful?
      - Screening for all possible adversities that a person may face is impossible.
      - Can instead promote the practice of “universal” trauma precautions given the ubiquity of traumatic exposures (90% of US adults have had exposure to at least one traumatic event).
      - In addition, most physicians are not trained in trauma assessment/treatment. Therefore, delving into patients’ specific traumas is not always appropriate. Can ask about stressors broadly and encourage connection with mental health providers. Trauma-informed care is a “team sport.”
      - Can consider specific stressor screening in distinct populations, i.e., screening for military-specific stressors in military populations. For example, for military-connected adolescents, can be incorporated into the HEADSS exam: H: “Is anybody in the home currently deployed”, E: “How long have you been at your current school?”
      - Ultimate use of the central tenet of trauma-informed care can be a great “first step” to practicing with a trauma-informed lens. Suspending judgment and viewing “difficult” behaviors or health diagnoses through a lens of trauma.
   2. What can be helpful about addressing adversity/trauma in clinical settings?
      - Referral to resources of support:
        1. Military OneSource
        2. Embedded mental health resources within clinics
        3. Telemynd
      - Opportunity to validate patients’ experience, educate on the link between stress and health, and collaborate on treatment plans
      - Opportunity to highlight strengths, for children/adolescents, to foster positive childhood experiences (Optional review for additional information: [Tufts HOPE – Healthy Outcomes from Positive Experiences](https://positiveexperience.org/))

**Case 1:** The intent of this case inclusion is to drive home the use of “universal trauma precautions” and the application of the central tenet of trauma-informed care.

A 19 year-old man, active-duty service member. He lives with his 18-year-old spouse and they are currently expecting their first child. He is an active smoker, 1 pack a day, and asks for suggestions on how he may quit nicotine, as he wants to be healthier for his family.

This case offers an opportunity to review the discussion points from above. The slides include specific data linking both adolescent pregnancy and tobacco use to a history of traumatic exposures. Can emphasize for students that we may not know an individual’s specific history, but can see their behaviors and diagnoses through a lens of trauma to help to obtain a holistic picture. This can be an entry point to discussion of the impact of stress on health, validation, collaboration, and referral to resources of support.

References:

1. About the CDC-Kaiser Ace Study. Centers for Disease Control and Prevention. Accessed August 6, 2024. <https://www.cdc.gov/violenceprevention/aces/about.html>
2. *What is Trauma-Informed Care?* [YouTube]. Center for Health Care Strategies; 2019. Accessed August 6, 2024. <https://youtu.be/fWken5DsJcw>
3. *Health Care Provider's Guide to Trauma-Informed Care*. Psychological Health Center of Excellence. Updated March 2018. Accessed August 6, 2024. <https://jko.jten.mil/courses/CTIP_healthcare_toolkit/courseFiles/ContentPages/CoursePages/resources/PHCoE_TraumaProviderBrochure_v0.9_Final%20508_07MAR2018_.pdf>

Lamson A, Richardson N, Cobb E. The health and readiness of service members: ACEs to PACEs. *Mil Med*. 2020;185(suppl 1):348-354. <https://doi.org/10.1093/milmed/usz197>
