# Supplementary material for: The Application of Trauma-Informed Care to Health Care for Military-Connected Individuals
Source: MedEdPORTAL. 2024 Nov 5;20:11466. doi: 10.15766/mep_2374-8265.11466 (PMC11534622; doi:10.15766/mep_2374-8265.11466)
Supplement: Supplementary file 1 — Slide Set.pptxPresession Message.docxPre-Post Evaluation.docxFacilitator Guide.docx [file mep_2374-8265.11466-s001.zip › B. Presession Message.docx]

Appendix B: Presession Message

**Instructions:** This pre-session message should be sent to learners 1 week prior to the scheduled workshop. The survey should take <5 minutes to complete and the option pre-reading should take 15-20 minutes to complete.

**Trauma Informed Care Session**: Information and Objectives

**Please join the session on your own, individual computer, as we will be utilizing breakout rooms.**

In order to ensure this session meets learner needs, we want to evaluate its efficacy. Please **take 2-5 minutes to complete this pre-test**(Appendix C).

Your participation is this pre-test is completely voluntary. The responses are confidential and will not impact your grade in any way. No identifying information will be collected and your name will not be associated with your responses.

**Optional Pre-Reading: (15-20 minutes)**

- About the CDC-Kaiser ACE Study^[[1]](#footnote-1)^ <https://www.cdc.gov/violenceprevention/aces/about.html>

*Informational website with summarized content regarding the the original ACES study, with linked information about the study questionnaires, data and statistics, and major findings.*

- [What](https://www.youtube.com/watch?v=fWken5DsJcw) is Trauma-Informed Care?^[[2]](#footnote-2)^

<https://youtu.be/fWken5DsJcw>

*Informational video from the Center for Health Care Strategies, Trauma-Informed Care Implementation Research Center.*

- [Health](https://www.qmo.amedd.army.mil/ptsd/PHCoE_TraumaProviderBrochure_v0.9_Final%20508_07MAR2018_.pdf) Care Provider’s Guide to Trauma-Informed Care^[[3]](#footnote-3)^

<https://jko.jten.mil/courses/CTIP_healthcare_toolkit/courseFiles/ContentPages/CoursePages/resources/PHCoE_TraumaProviderBrochure_v0.9_Final%20508_07MAR2018_.pdf>

*A 2-page informational guide on the principles of trauma-informed care as they could apply to military service members and veterans, with a list of pertinent resources.*

**Session Objectives:**

1. Define trauma and toxic stress and their connection to health
2. Describe the importance and components of a trauma-informed approach to care
3. Identify the relevance of trauma-informed care to military medicine

1. About the CDC-Kaiser Ace Study. Centers for Disease Control and Prevention, Violence Prevention. Updated April 6 2021. Accessed August 6, 2024.  Available at: https://www.cdc.gov/violenceprevention/aces/about.html [↑](#footnote-ref-1)
2. What is Trauma-Informed Care? Center for Health Care Strategies. Trauma-Informed Care Implementation Resource Center. Accessed August 6, 2024. Available at: https://youtu.be/fWken5DsJcw [↑](#footnote-ref-2)
3. Health Care Provider’s Guide to Trauma-Informed Care. Psychological Health Center of Excellence. Updated March 2018. Accessed August 6, 2024. Available at: https://jko.jten.mil/courses/CTIP_healthcare_toolkit/courseFiles/ContentPages/CoursePages/resources/PHCoE_TraumaProviderBrochure_v0.9_Final%20508_07MAR2018_.pdf [↑](#footnote-ref-3)
