# Supplementary material for: The Application of Trauma-Informed Care to Health Care for Military-Connected Individuals
Source: MedEdPORTAL. 2024 Nov 5;20:11466. doi: 10.15766/mep_2374-8265.11466 (PMC11534622; doi:10.15766/mep_2374-8265.11466)

## Slide 1
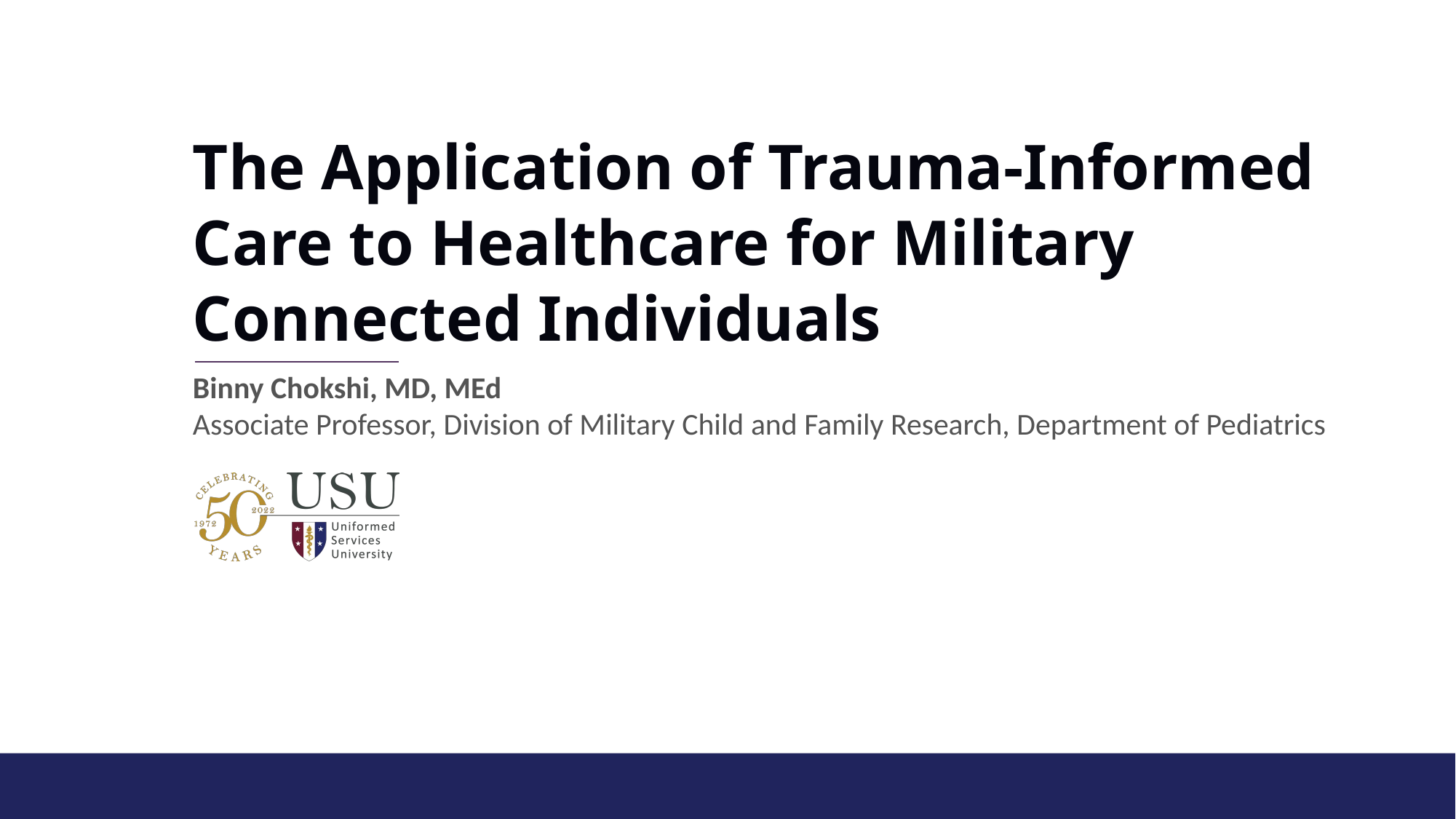

# The Application of Trauma-Informed Care to Healthcare for Military Connected Individuals
Binny Chokshi, MD, MEd
Associate Professor, Division of Military Child and Family Research, Department of Pediatrics

## Slide 2
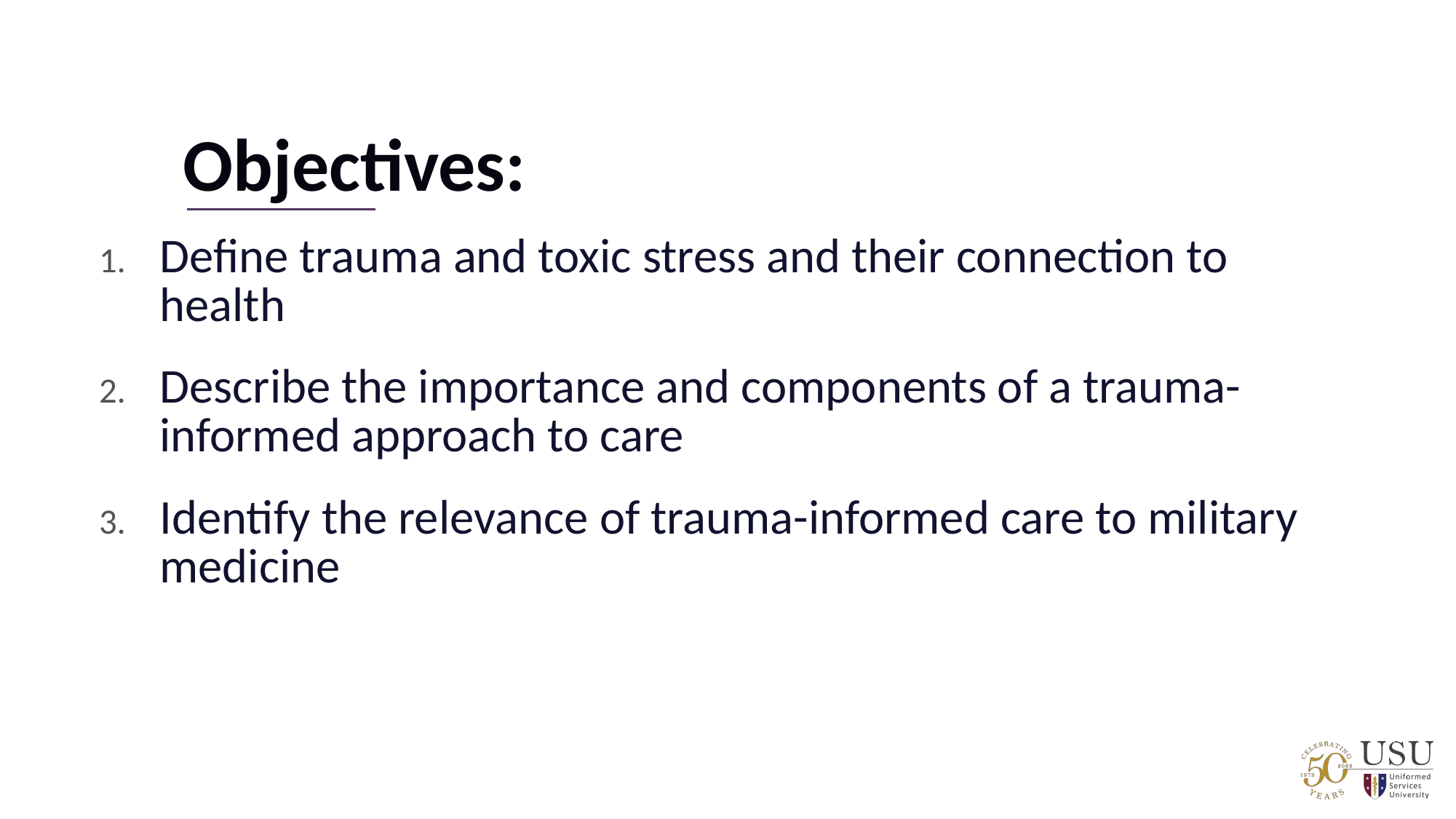

# Objectives:
Define trauma and toxic stress and their connection to health
Describe the importance and components of a trauma-informed approach to care
Identify the relevance of trauma-informed care to military medicine

## Slide 3
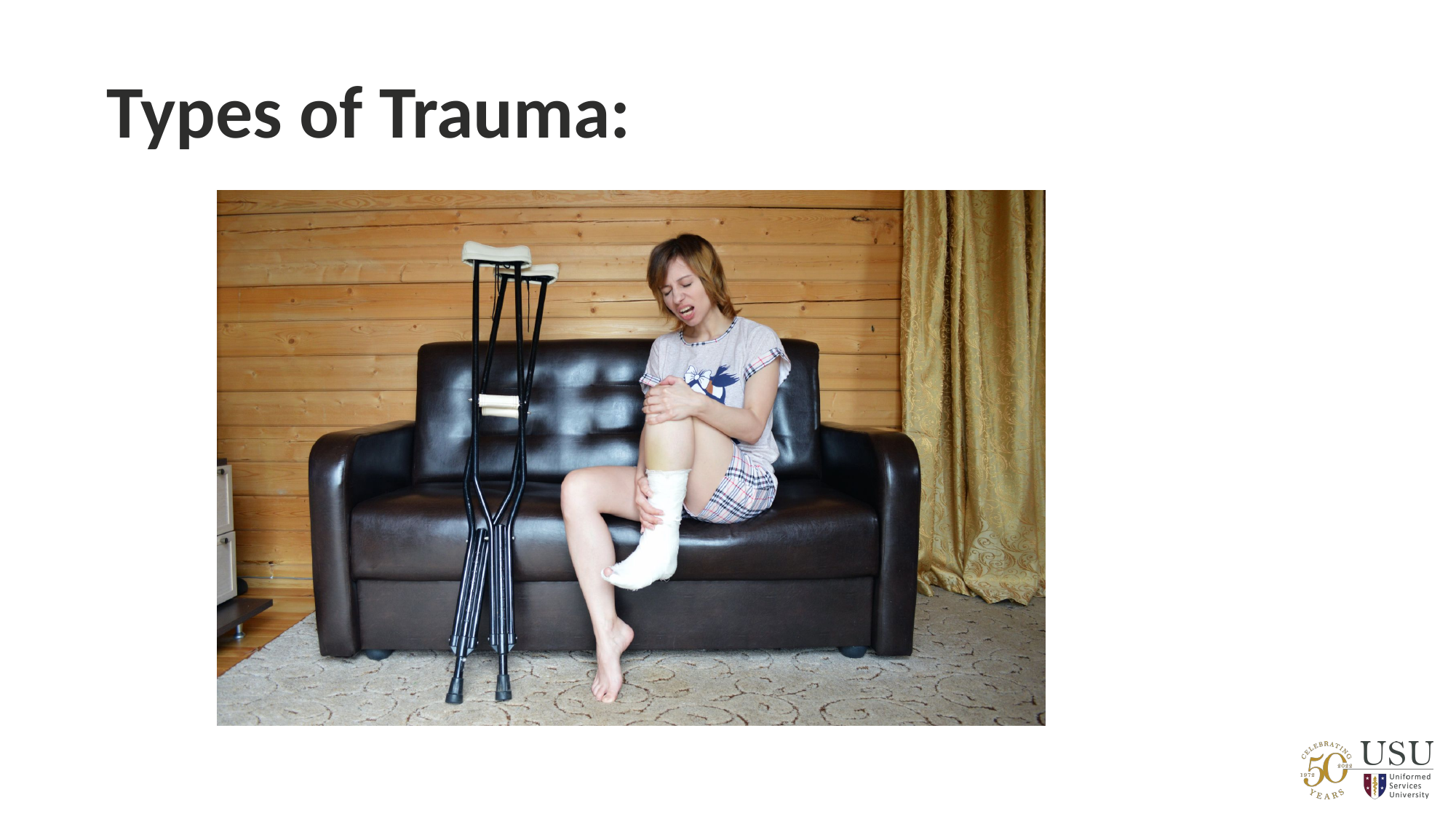

Types of Trauma:

## Slide 4
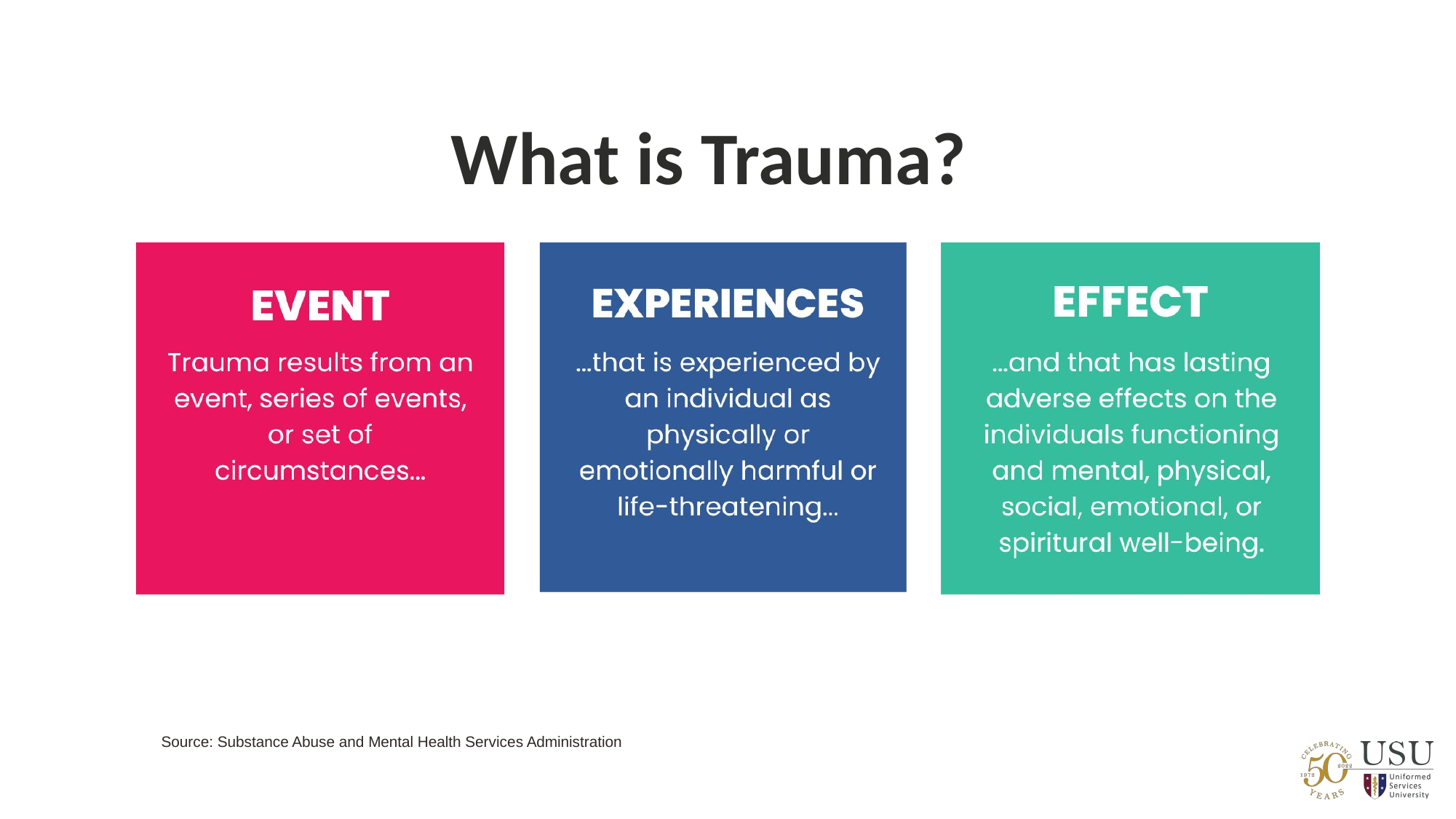

What is Trauma?
Source: Substance Abuse and Mental Health Services Administration

## Slide 5
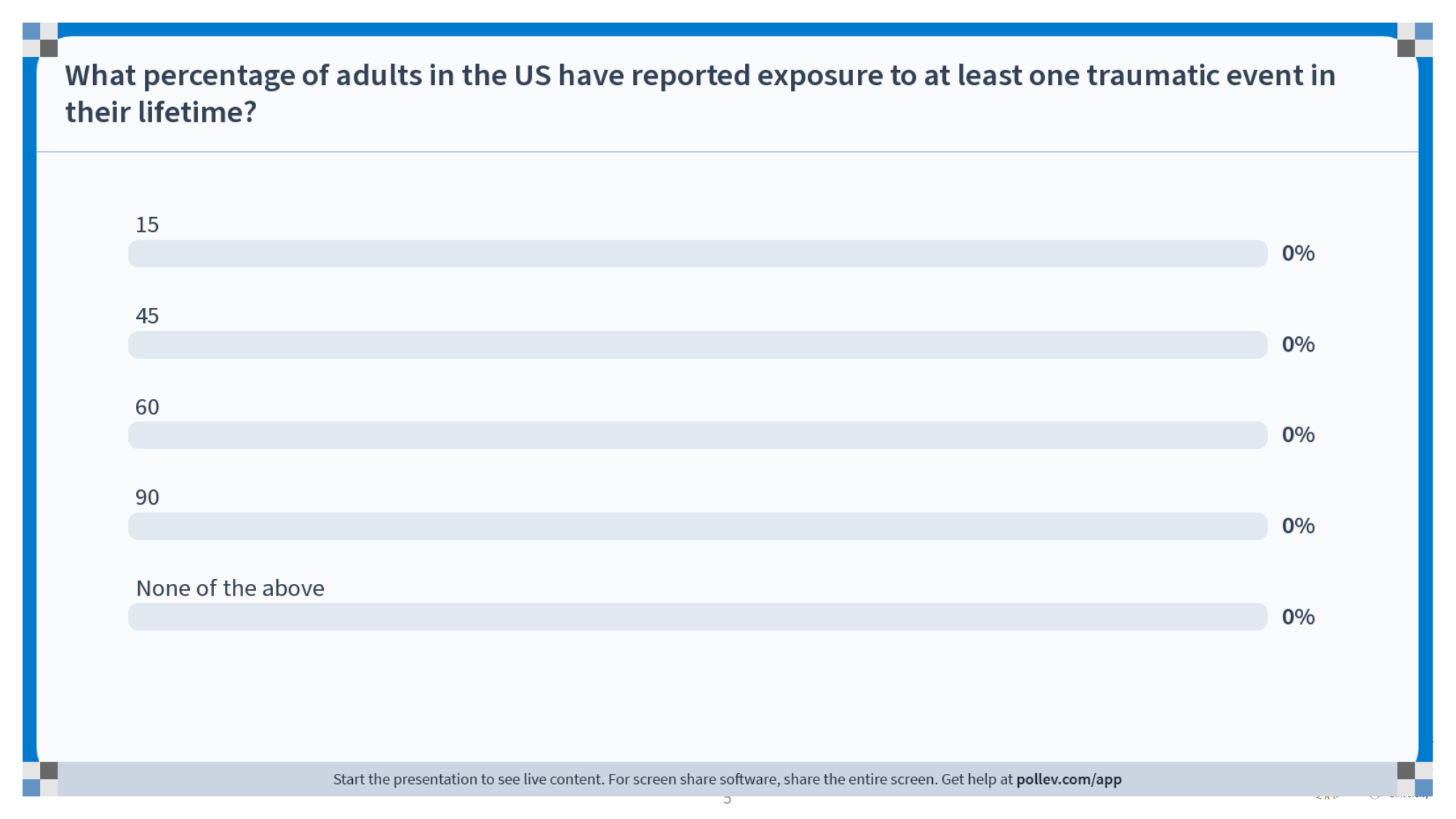

5

## Slide 6
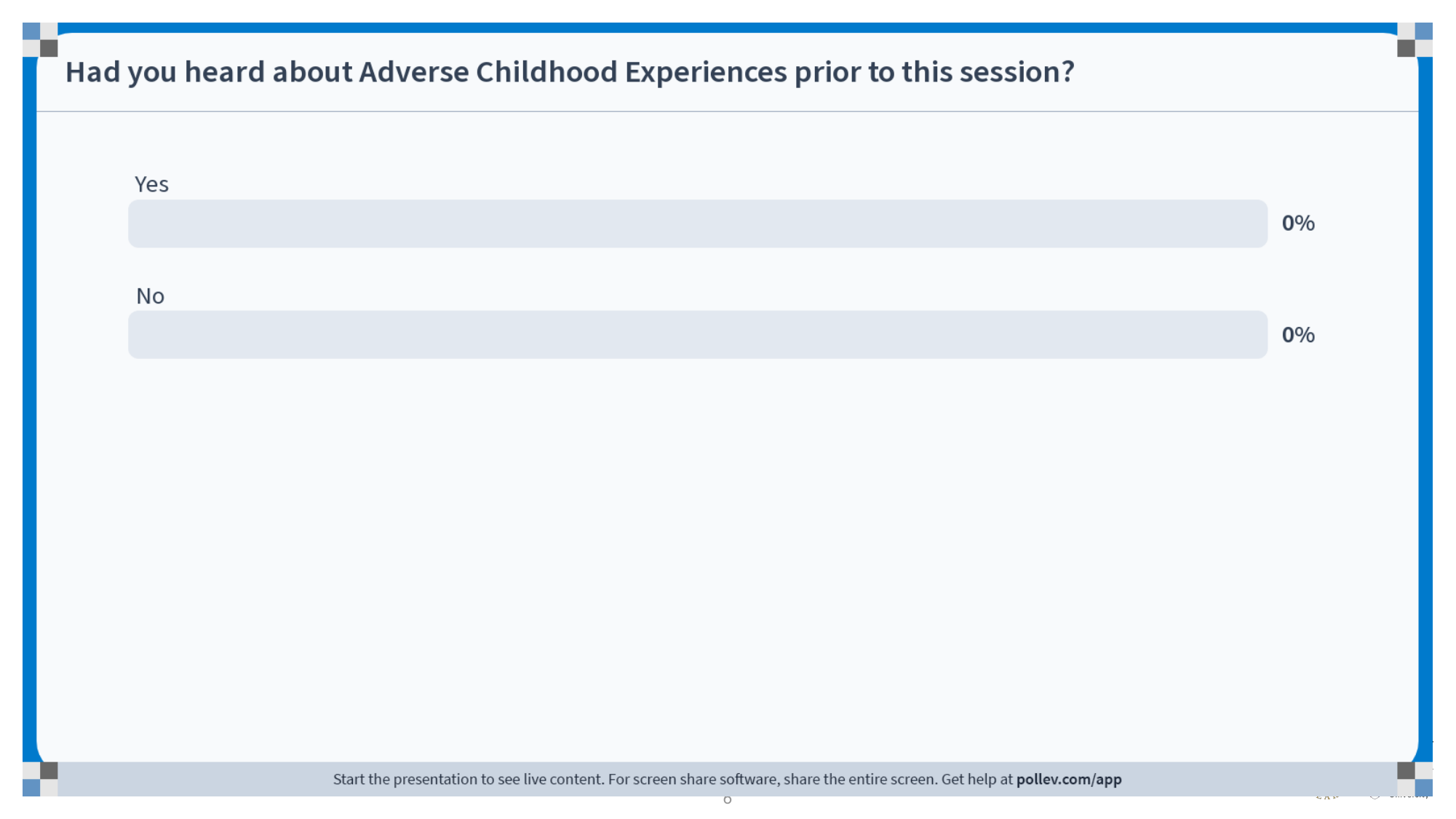

6

## Slide 7
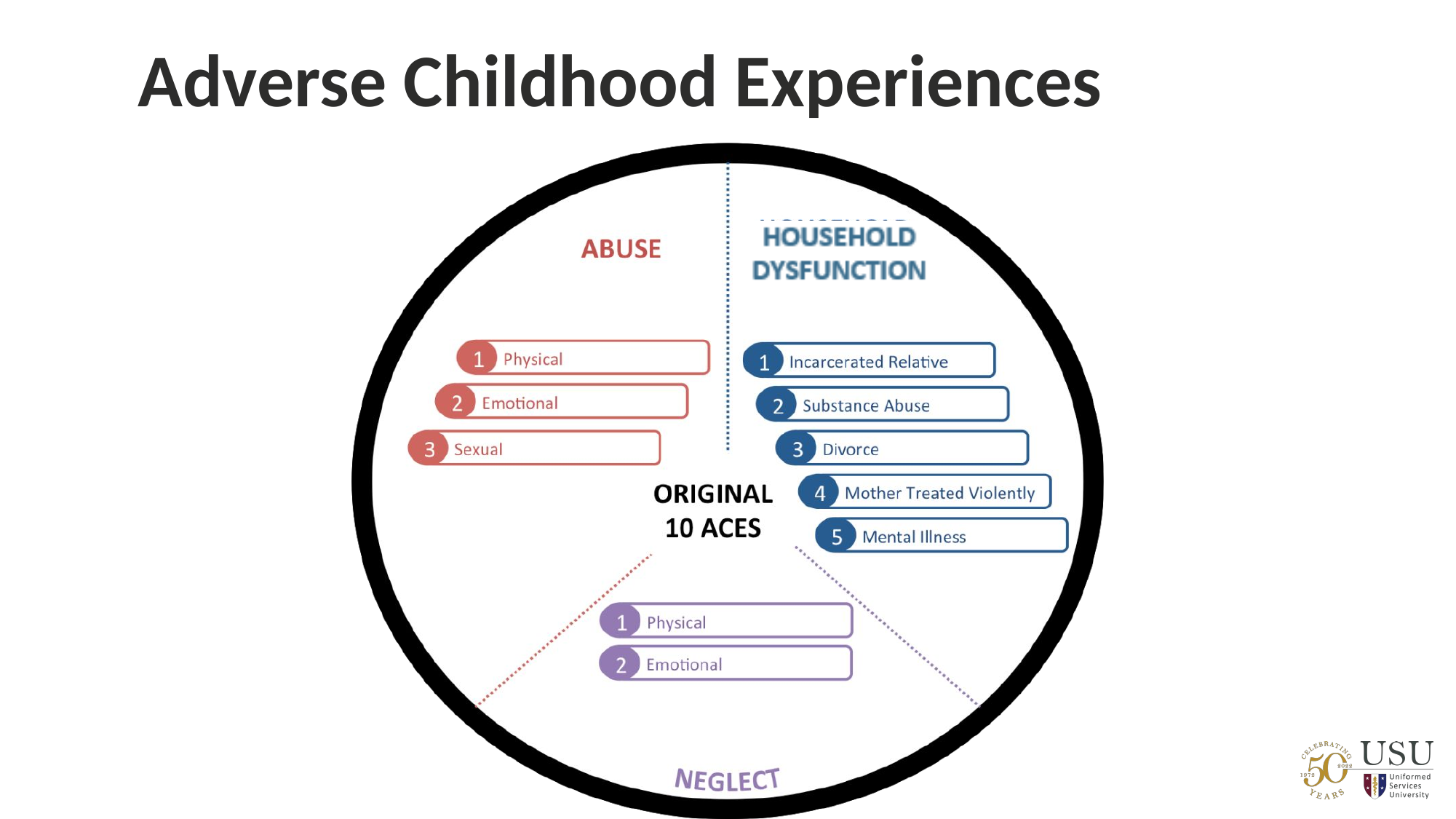

Adverse Childhood Experiences

## Slide 8
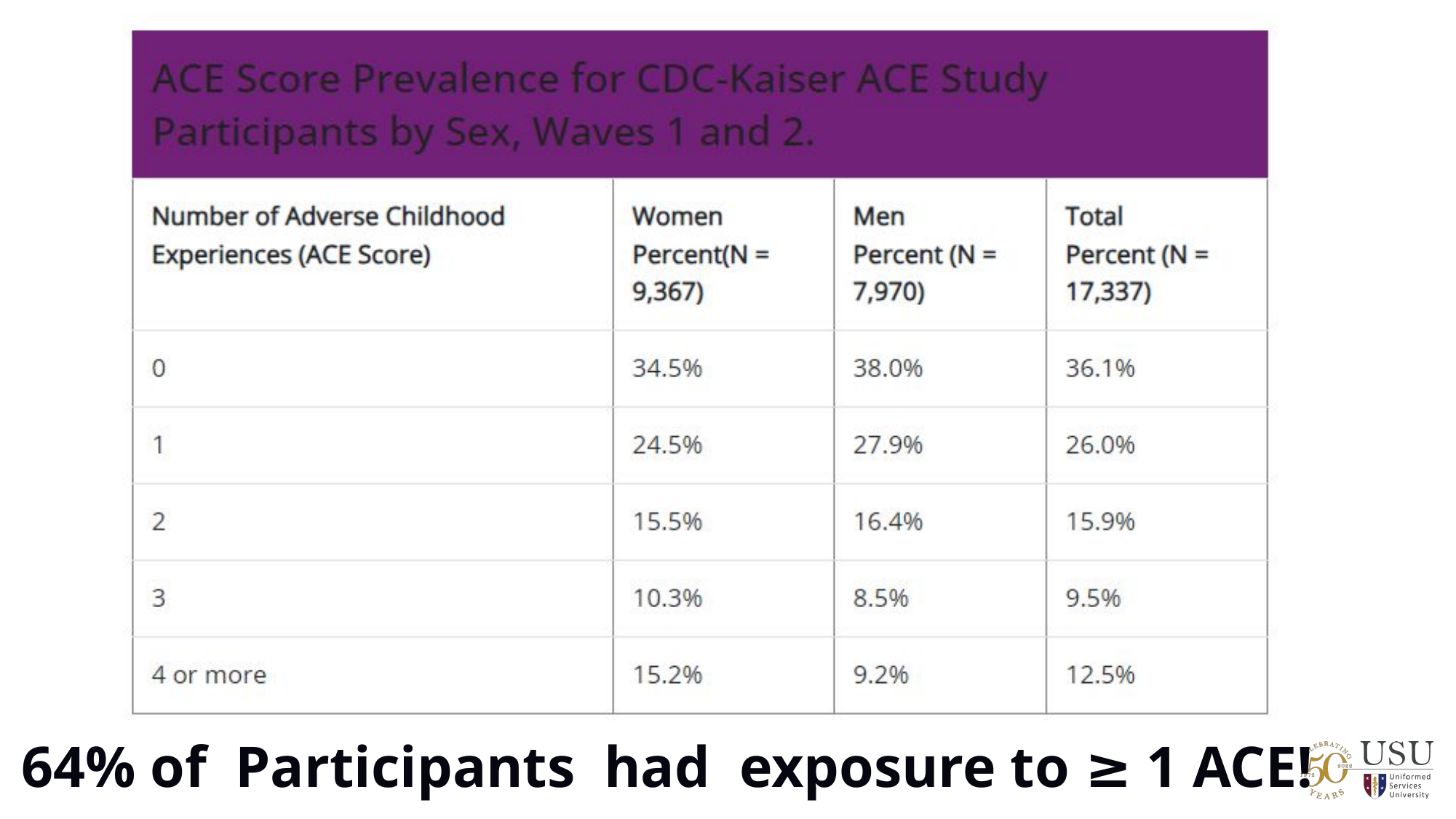

Adverse Childhood Experiences
64% of Participants had exposure to ≥ 1 ACE!

## Slide 9
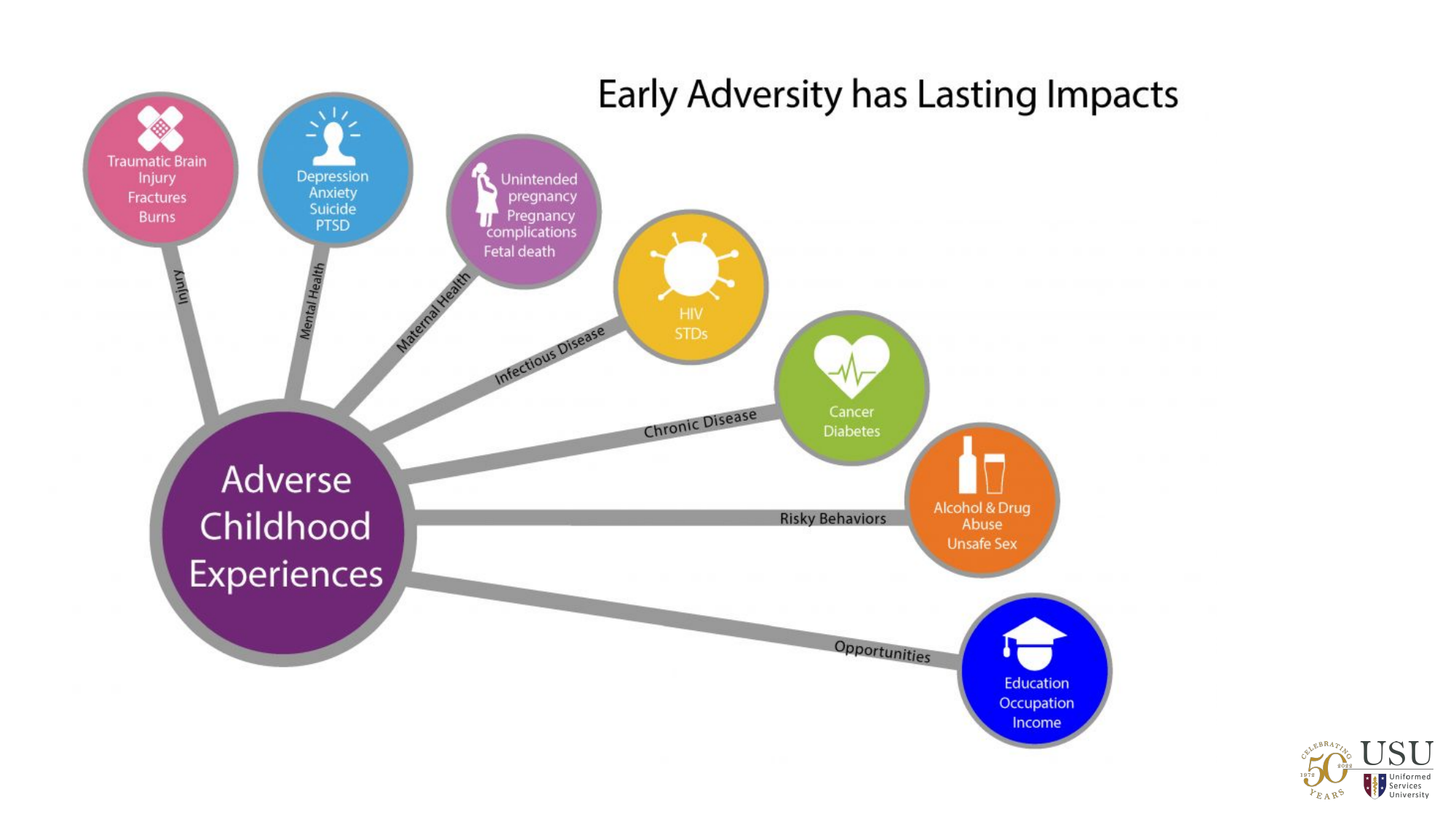

## Slide 10
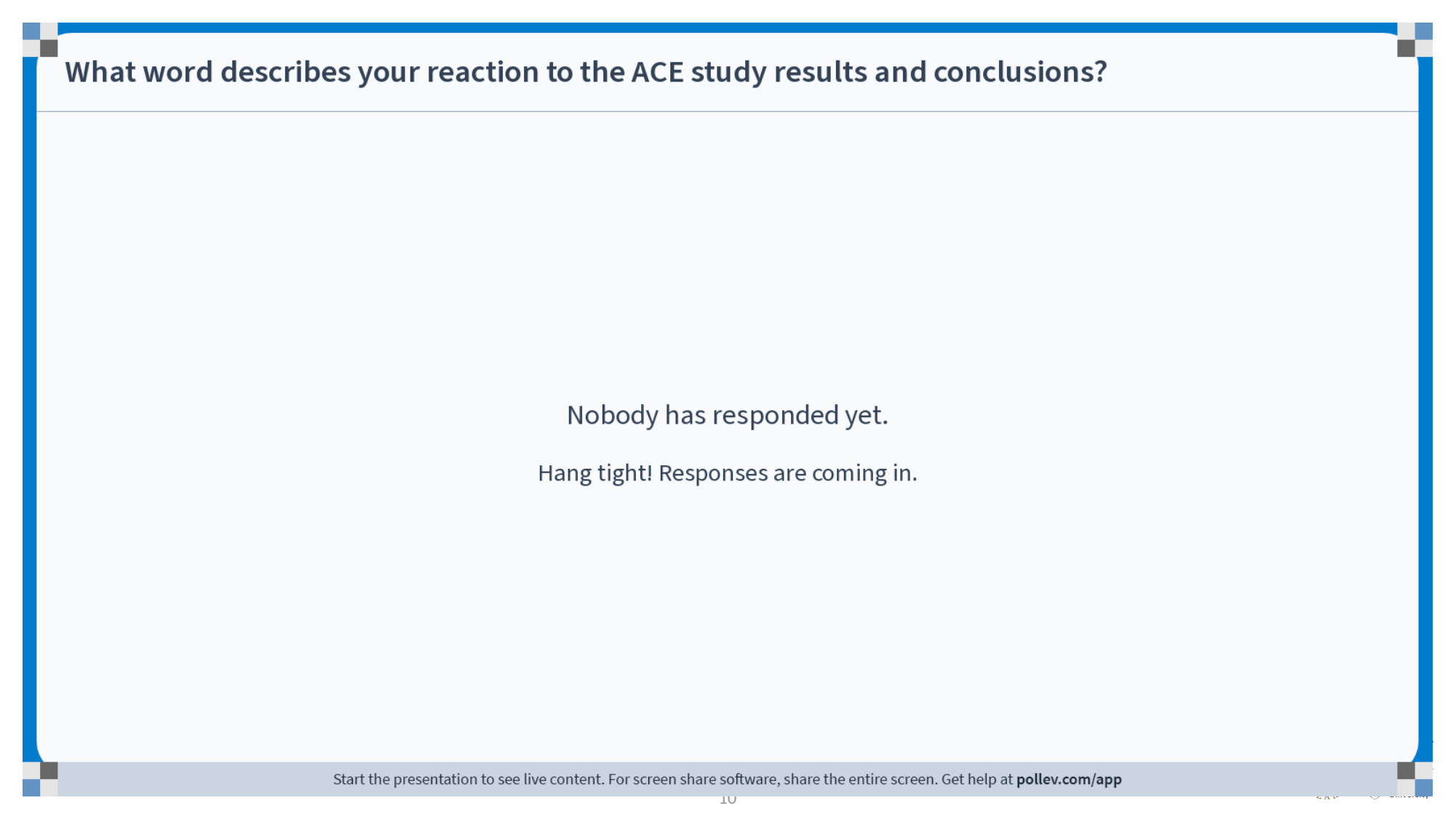

10

## Slide 11
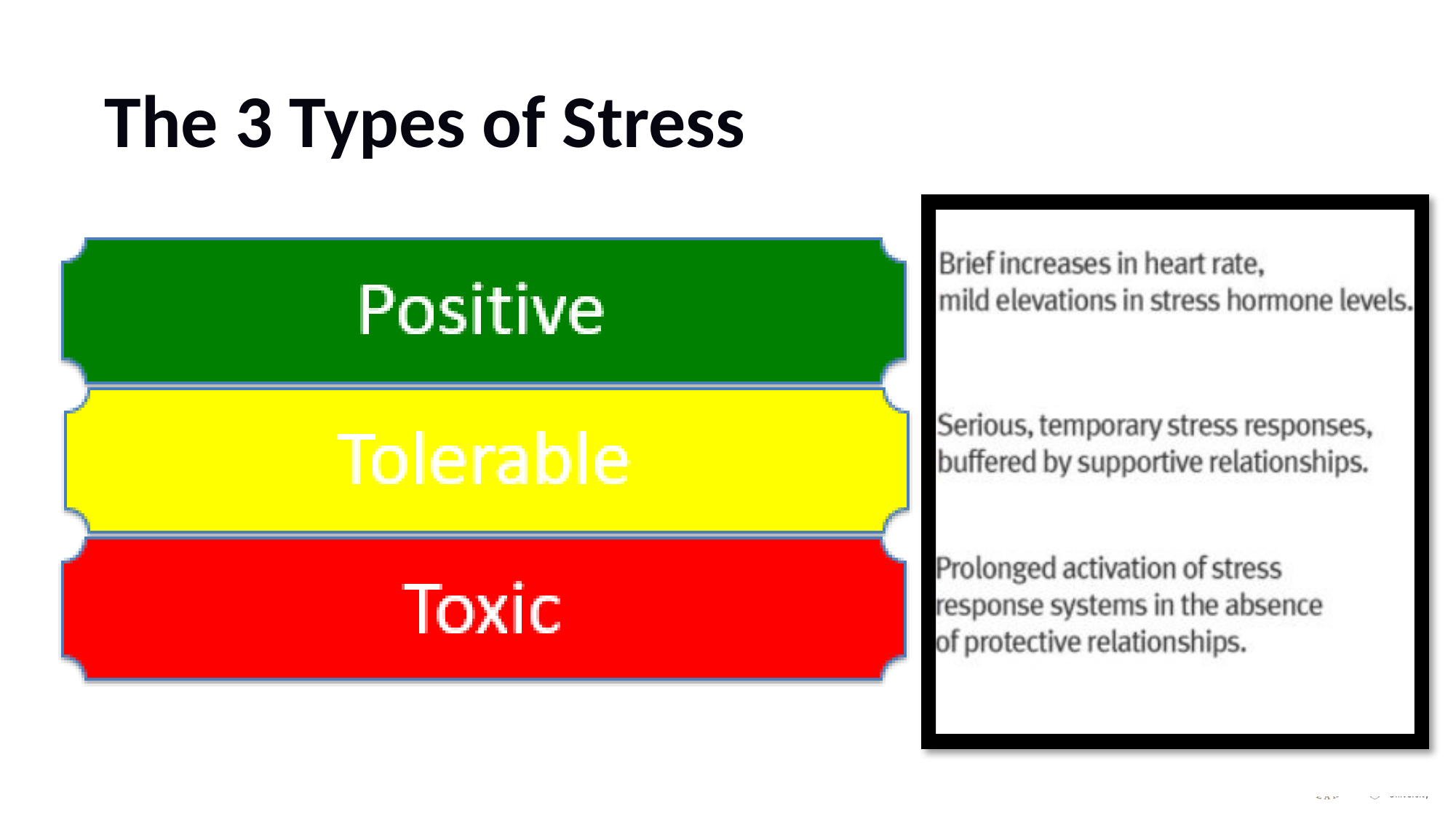

The 3 Types of Stress

## Slide 12
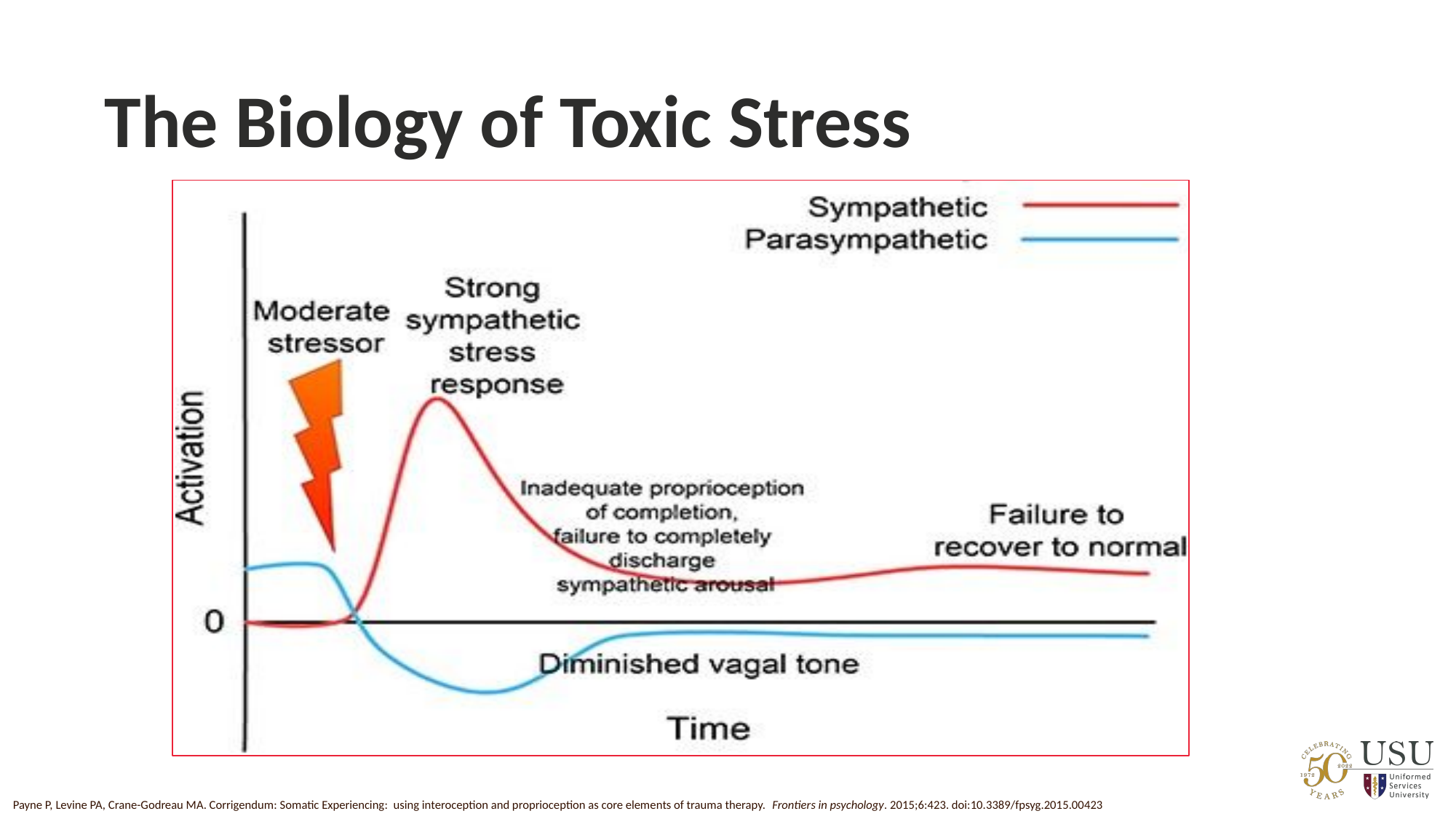

The Biology of Toxic Stress
Payne P, Levine PA, Crane-Godreau MA. Corrigendum: Somatic Experiencing: using interoception and proprioception as core elements of trauma therapy. Frontiers in psychology. 2015;6:423. doi:10.3389/fpsyg.2015.00423

## Slide 13
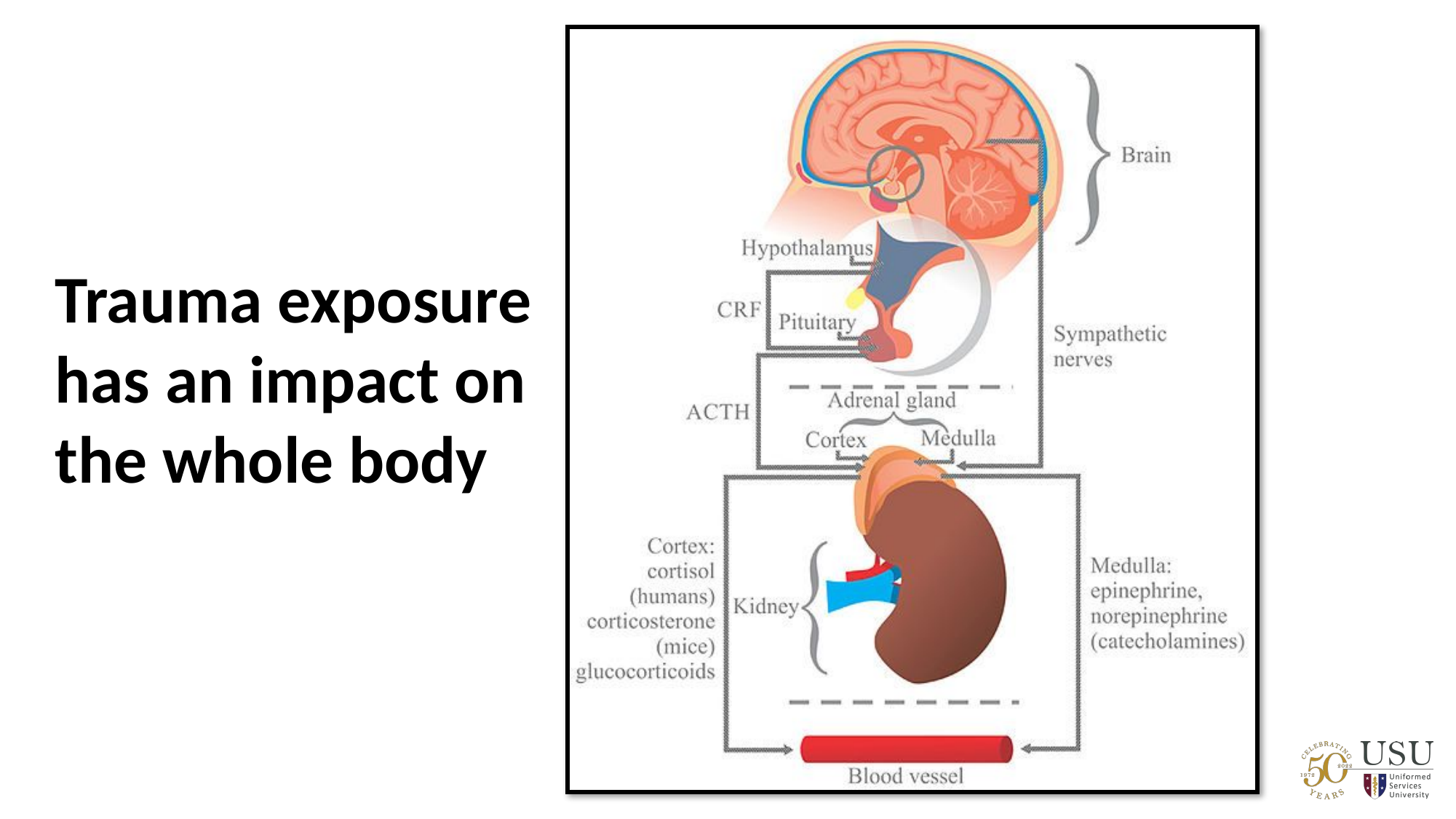

Trauma exposure has an impact on the whole body

## Slide 14
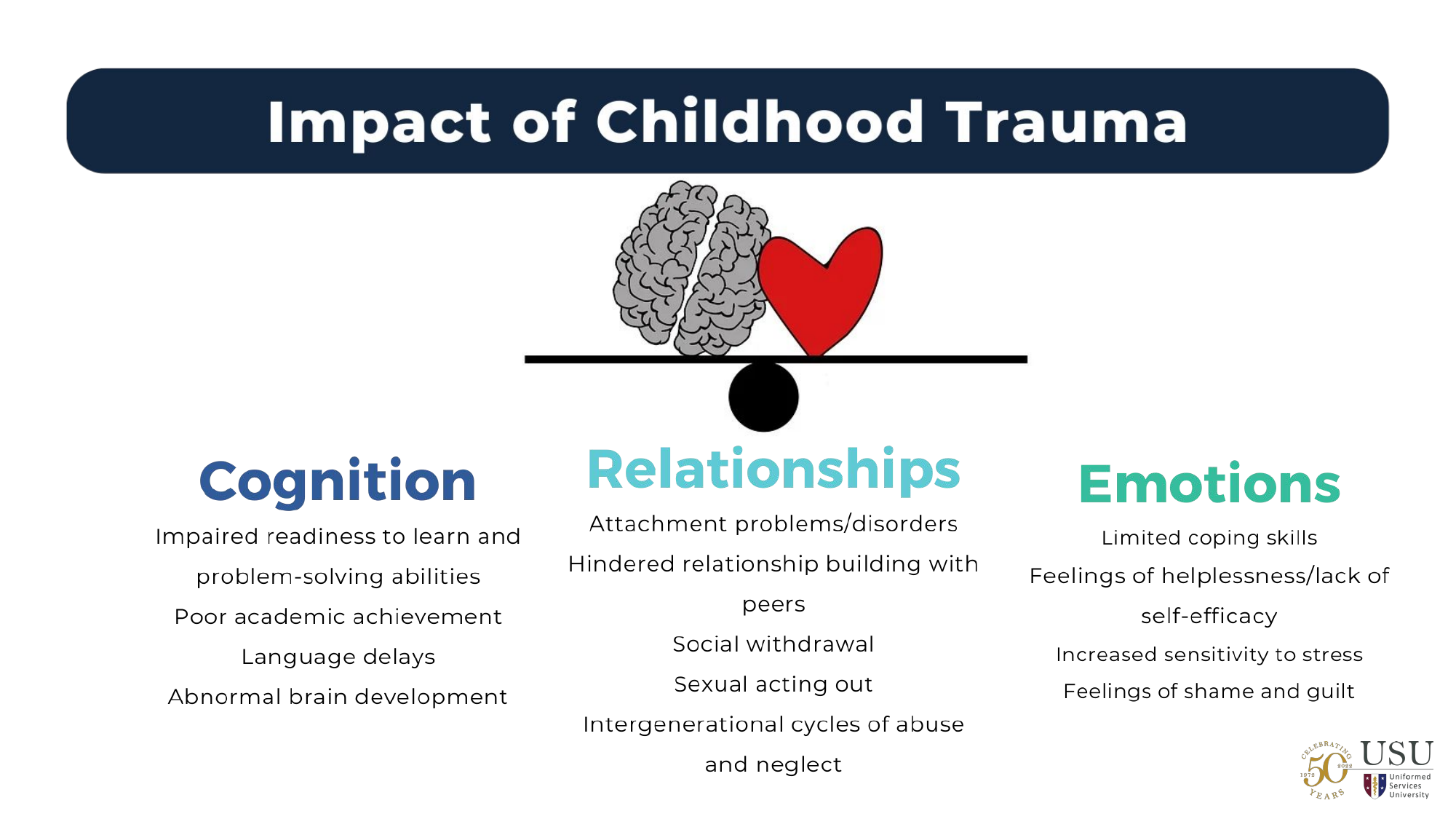

## Slide 15
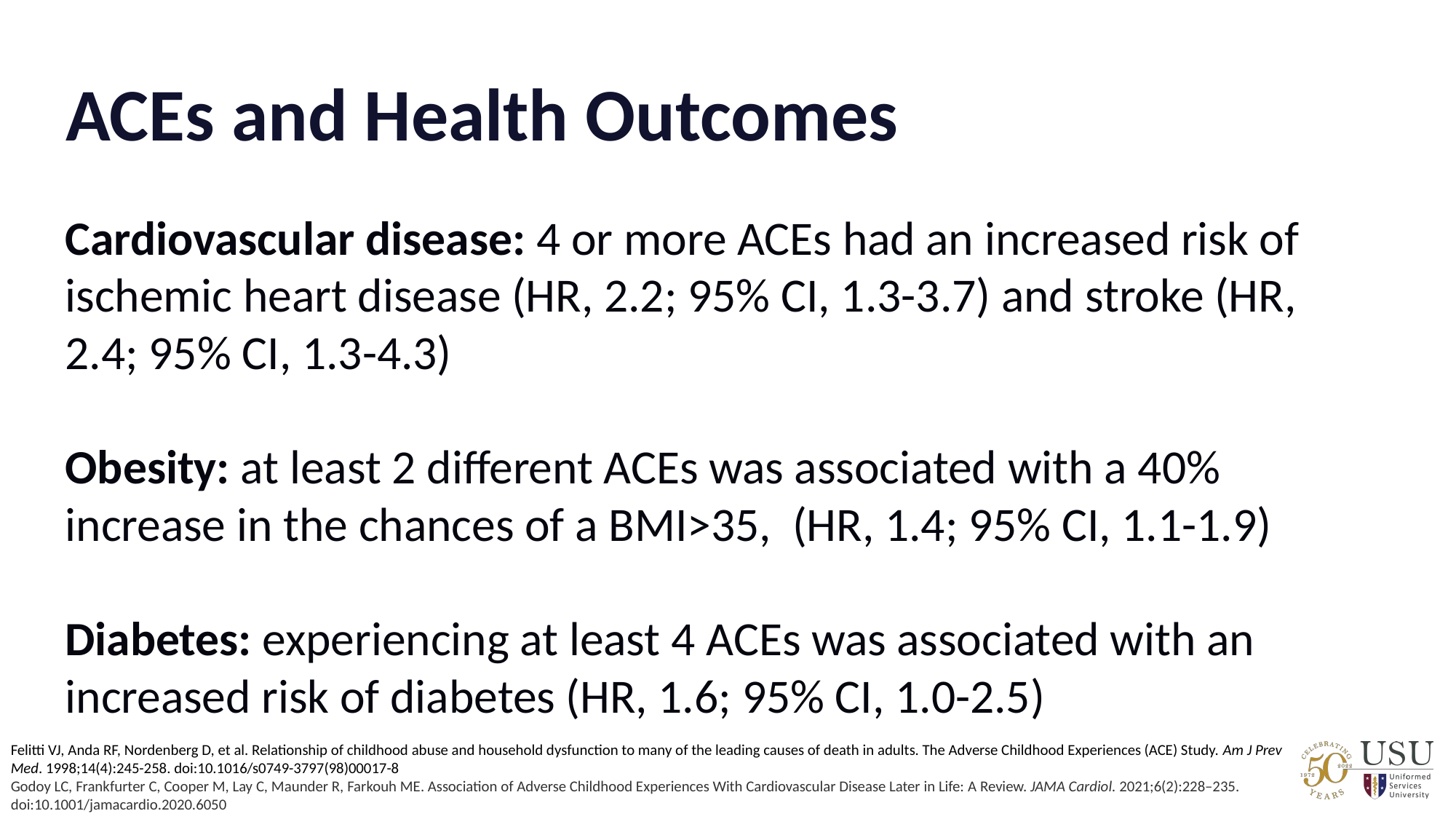

# ACEs and Health Outcomes
Cardiovascular disease: 4 or more ACEs had an increased risk of ischemic heart disease (HR, 2.2; 95% CI, 1.3-3.7) and stroke (HR, 2.4; 95% CI, 1.3-4.3)
Obesity: at least 2 different ACEs was associated with a 40% increase in the chances of a BMI>35, (HR, 1.4; 95% CI, 1.1-1.9)
Diabetes: experiencing at least 4 ACEs was associated with an increased risk of diabetes (HR, 1.6; 95% CI, 1.0-2.5)
Felitti VJ, Anda RF, Nordenberg D, et al. Relationship of childhood abuse and household dysfunction to many of the leading causes of death in adults. The Adverse Childhood Experiences (ACE) Study. Am J Prev Med. 1998;14(4):245-258. doi:10.1016/s0749-3797(98)00017-8
Godoy LC, Frankfurter C, Cooper M, Lay C, Maunder R, Farkouh ME. Association of Adverse Childhood Experiences With Cardiovascular Disease Later in Life: A Review. JAMA Cardiol. 2021;6(2):228–235. doi:10.1001/jamacardio.2020.6050

## Slide 16
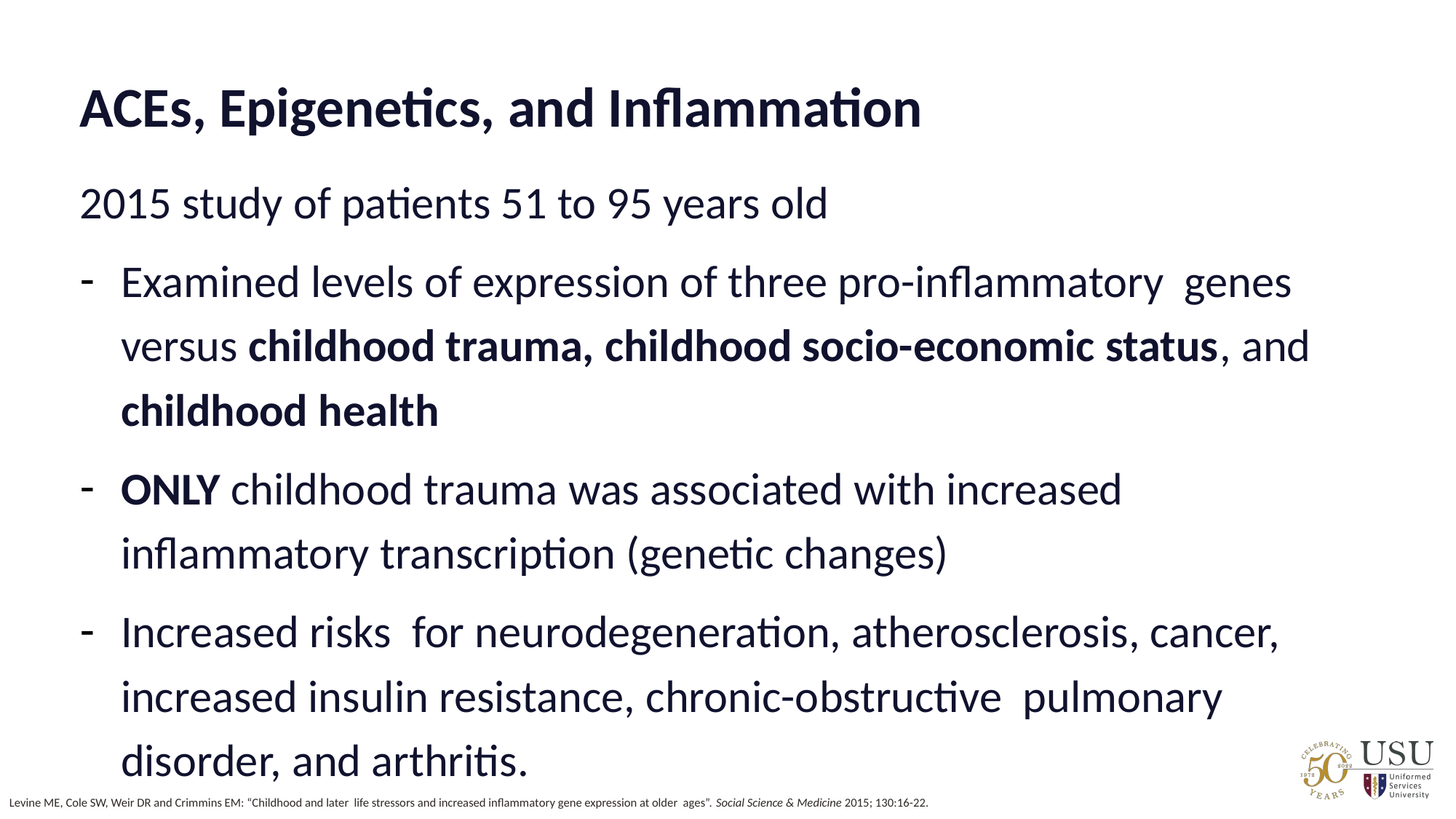

# ACEs, Epigenetics, and Inflammation
2015 study of patients 51 to 95 years old
Examined levels of expression of three pro-inflammatory genes versus childhood trauma, childhood socio-economic status, and childhood health
ONLY childhood trauma was associated with increased inflammatory transcription (genetic changes)
Increased risks for neurodegeneration, atherosclerosis, cancer, increased insulin resistance, chronic-obstructive pulmonary disorder, and arthritis.
Levine ME, Cole SW, Weir DR and Crimmins EM: “Childhood and later life stressors and increased inflammatory gene expression at older ages”. Social Science & Medicine 2015; 130:16-22.

## Slide 17
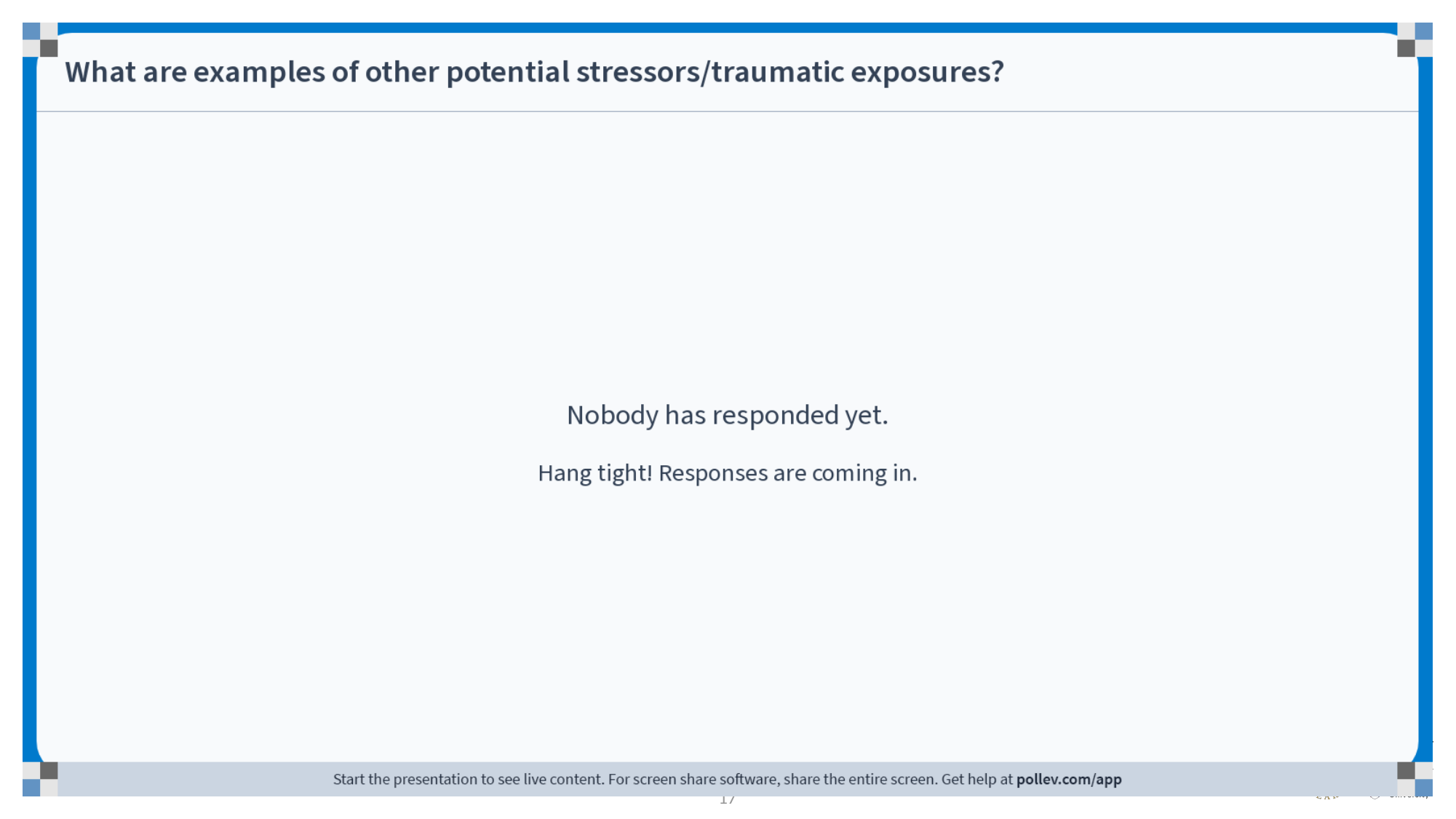

17

## Slide 18
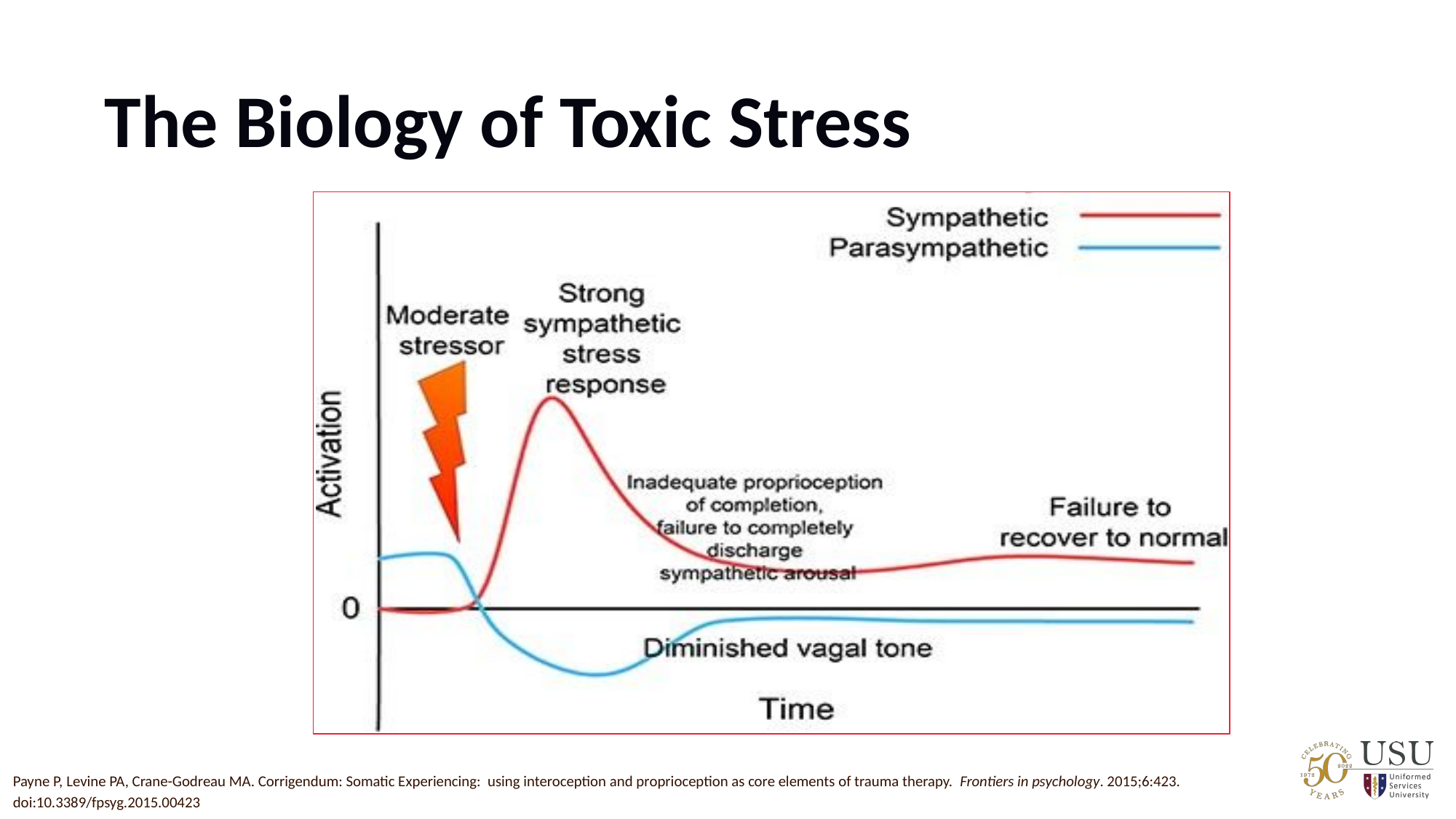

The Biology of Toxic Stress
Payne P, Levine PA, Crane-Godreau MA. Corrigendum: Somatic Experiencing: using interoception and proprioception as core elements of trauma therapy. Frontiers in psychology. 2015;6:423. doi:10.3389/fpsyg.2015.00423

## Slide 19
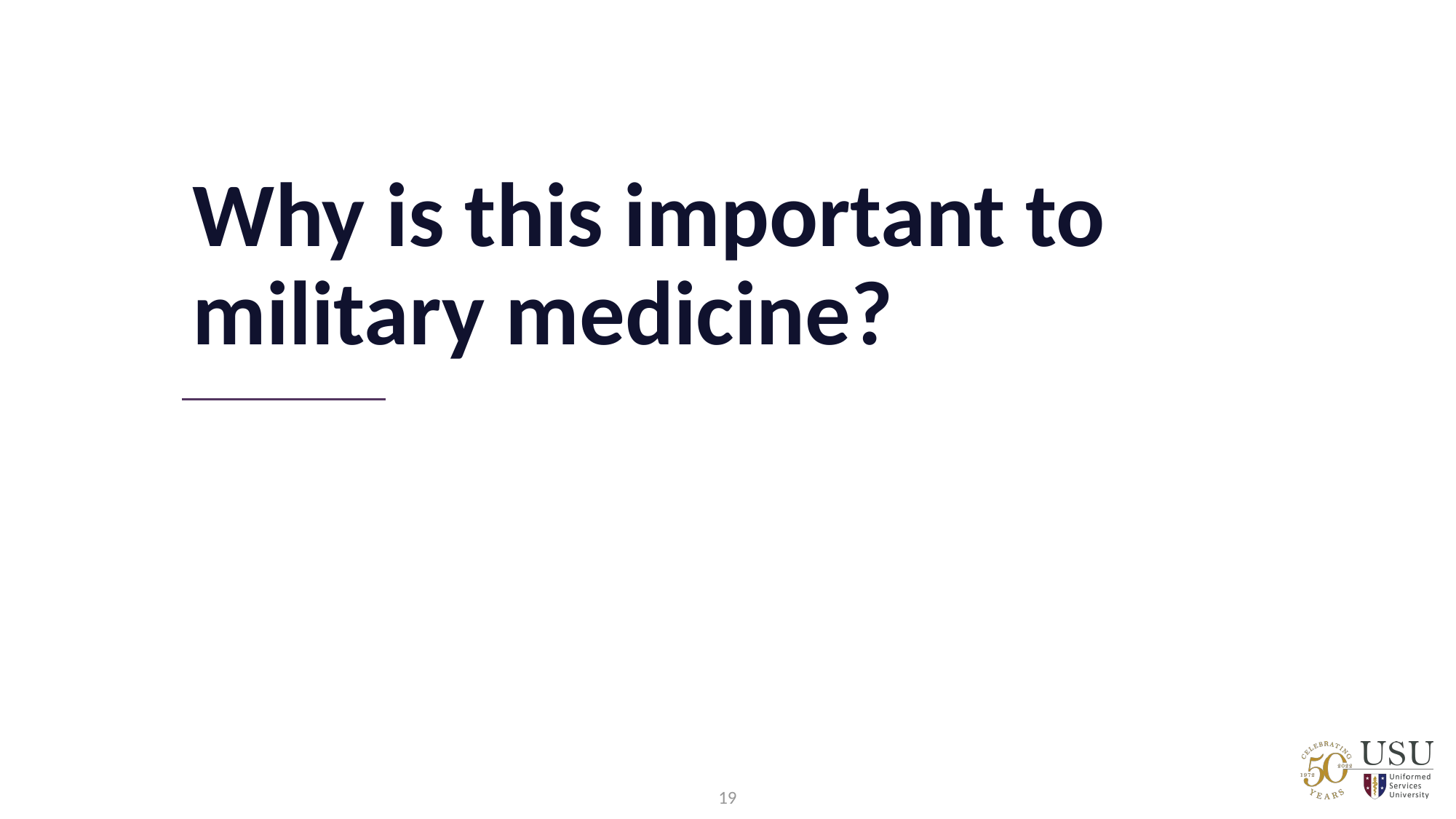

# Why is this important to military medicine?
19

## Slide 20
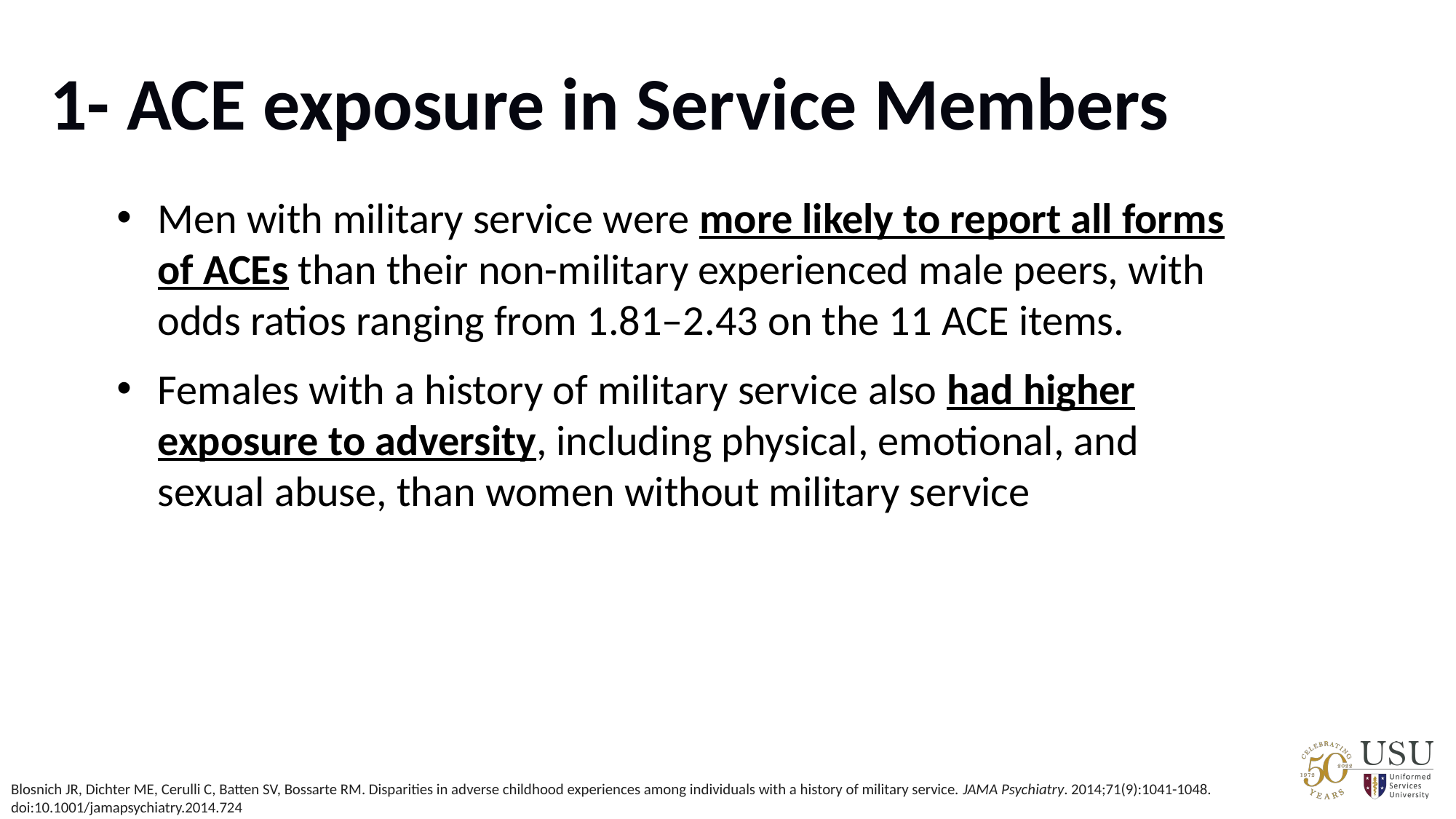

1- ACE exposure in Service Members
Men with military service were more likely to report all forms of ACEs than their non-military experienced male peers, with odds ratios ranging from 1.81–2.43 on the 11 ACE items.
Females with a history of military service also had higher exposure to adversity, including physical, emotional, and sexual abuse, than women without military service
Blosnich JR, Dichter ME, Cerulli C, Batten SV, Bossarte RM. Disparities in adverse childhood experiences among individuals with a history of military service. JAMA Psychiatry. 2014;71(9):1041-1048. doi:10.1001/jamapsychiatry.2014.724

## Slide 21
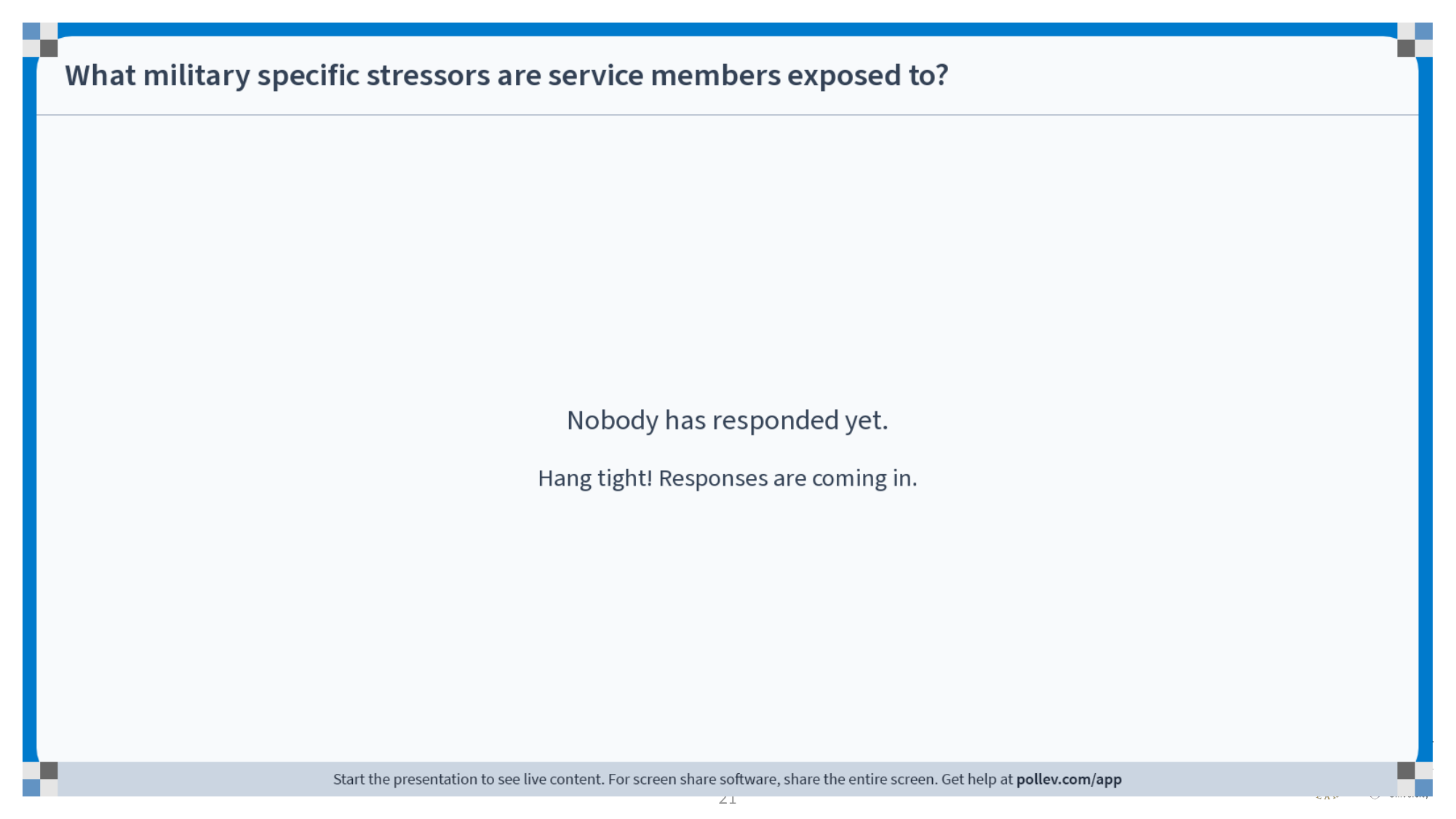

21

## Slide 22
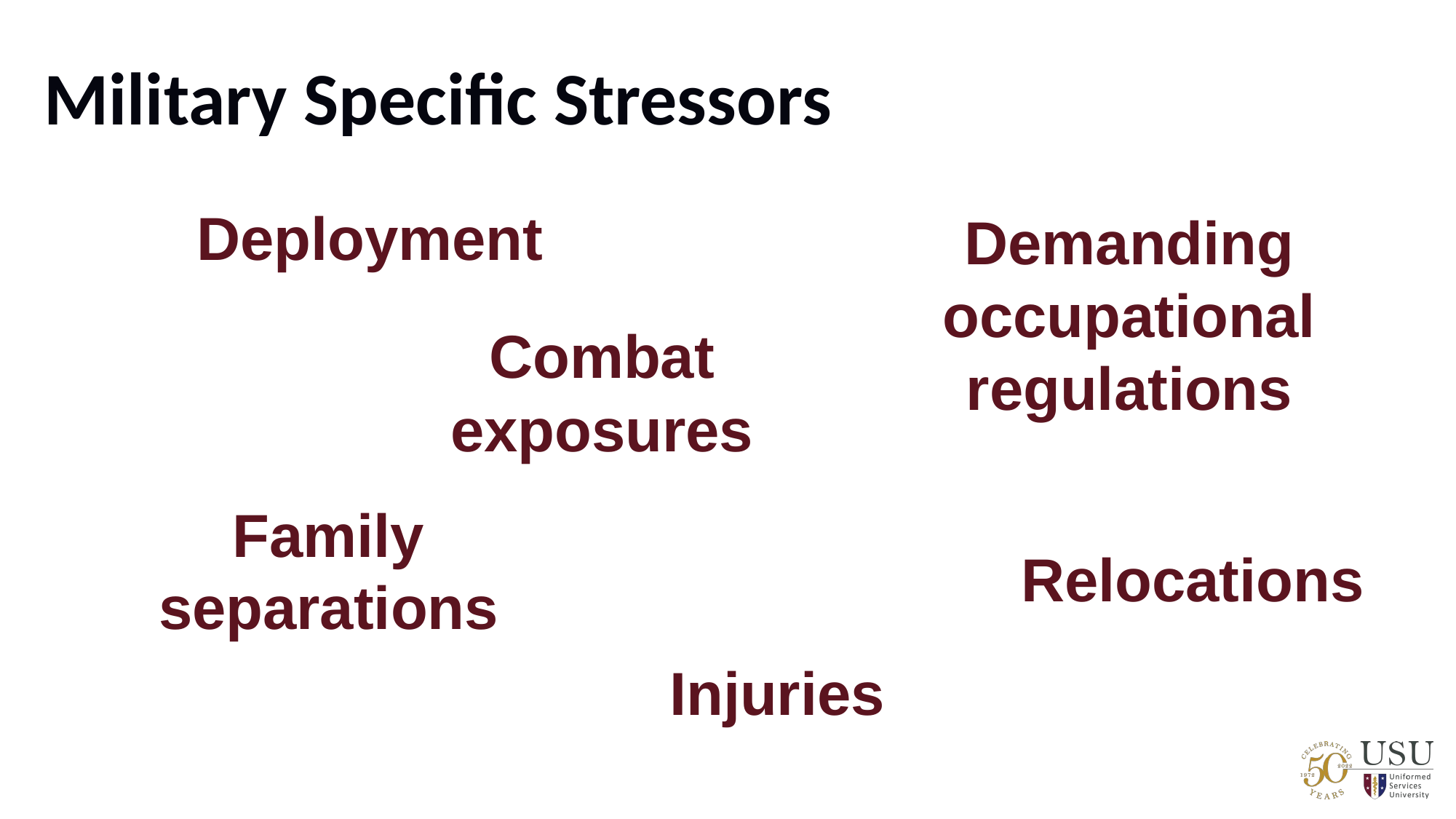

Military Specific Stressors
Deployment
Demanding occupational regulations
Combat exposures
Family separations
Relocations
Injuries

## Slide 23
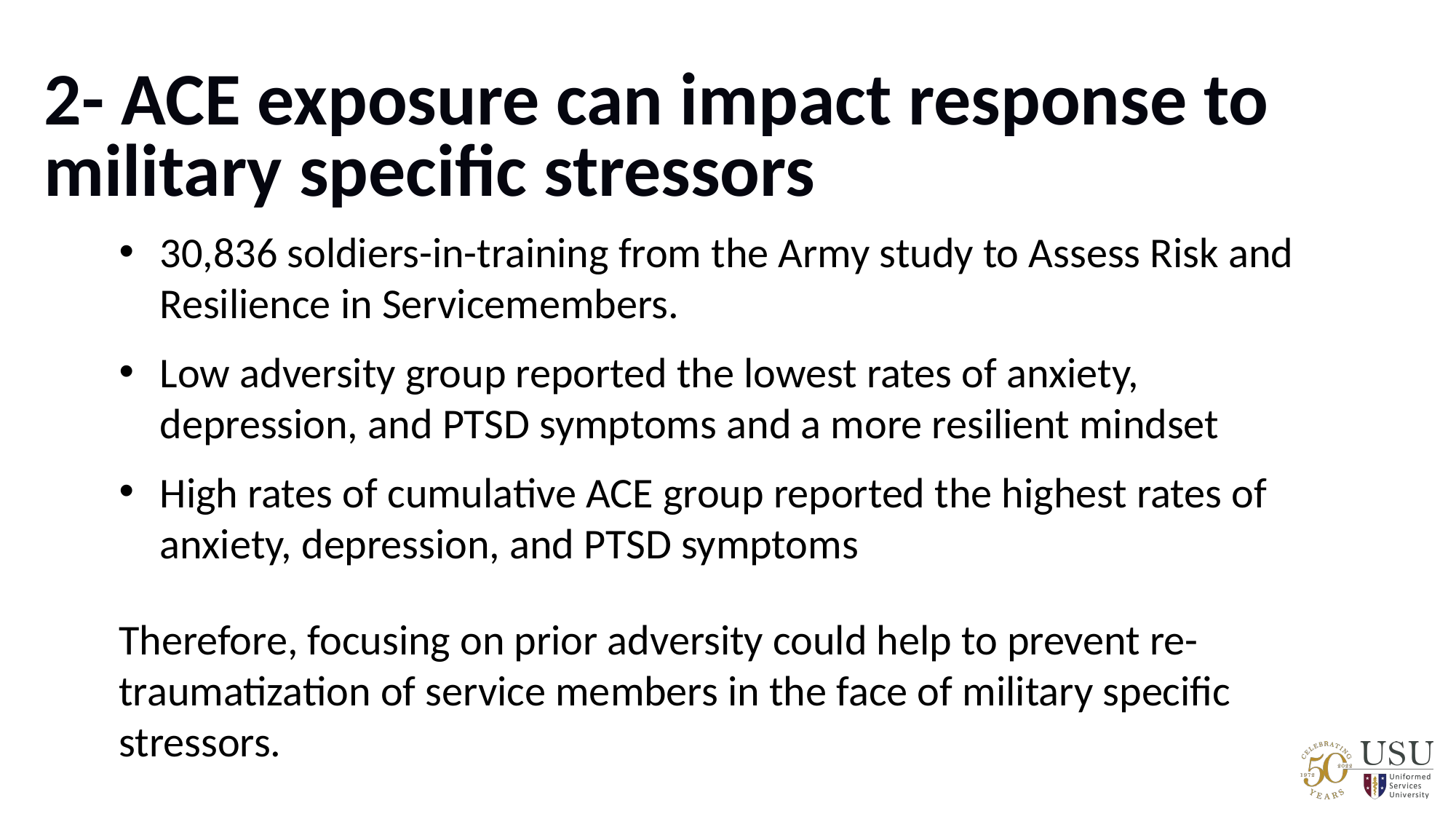

2- ACE exposure can impact response to military specific stressors
30,836 soldiers-in-training from the Army study to Assess Risk and Resilience in Servicemembers.
Low adversity group reported the lowest rates of anxiety, depression, and PTSD symptoms and a more resilient mindset
High rates of cumulative ACE group reported the highest rates of anxiety, depression, and PTSD symptoms
Therefore, focusing on prior adversity could help to prevent re-traumatization of service members in the face of military specific stressors.

## Slide 24
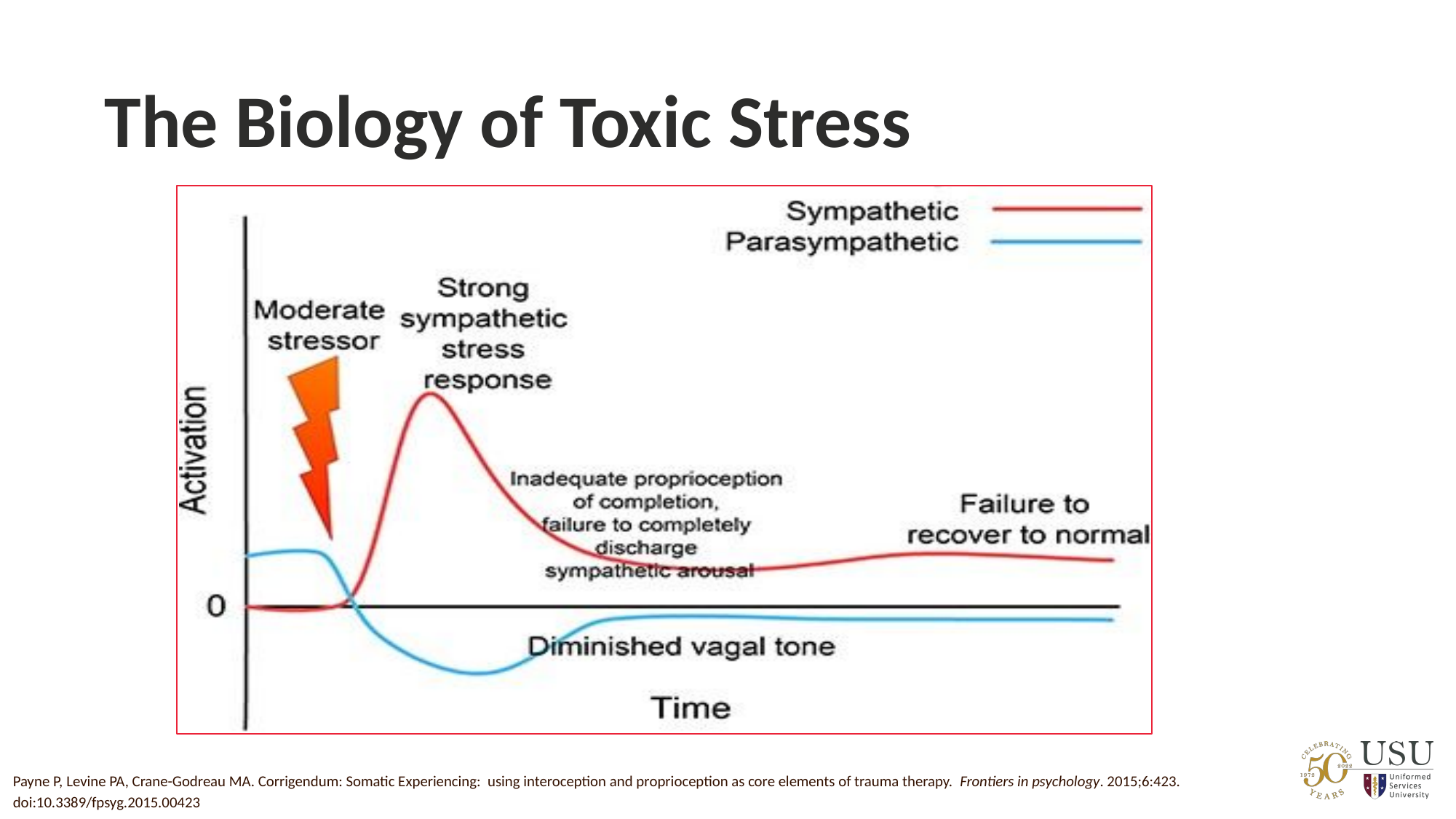

The Biology of Toxic Stress
Payne P, Levine PA, Crane-Godreau MA. Corrigendum: Somatic Experiencing: using interoception and proprioception as core elements of trauma therapy. Frontiers in psychology. 2015;6:423. doi:10.3389/fpsyg.2015.00423

## Slide 25
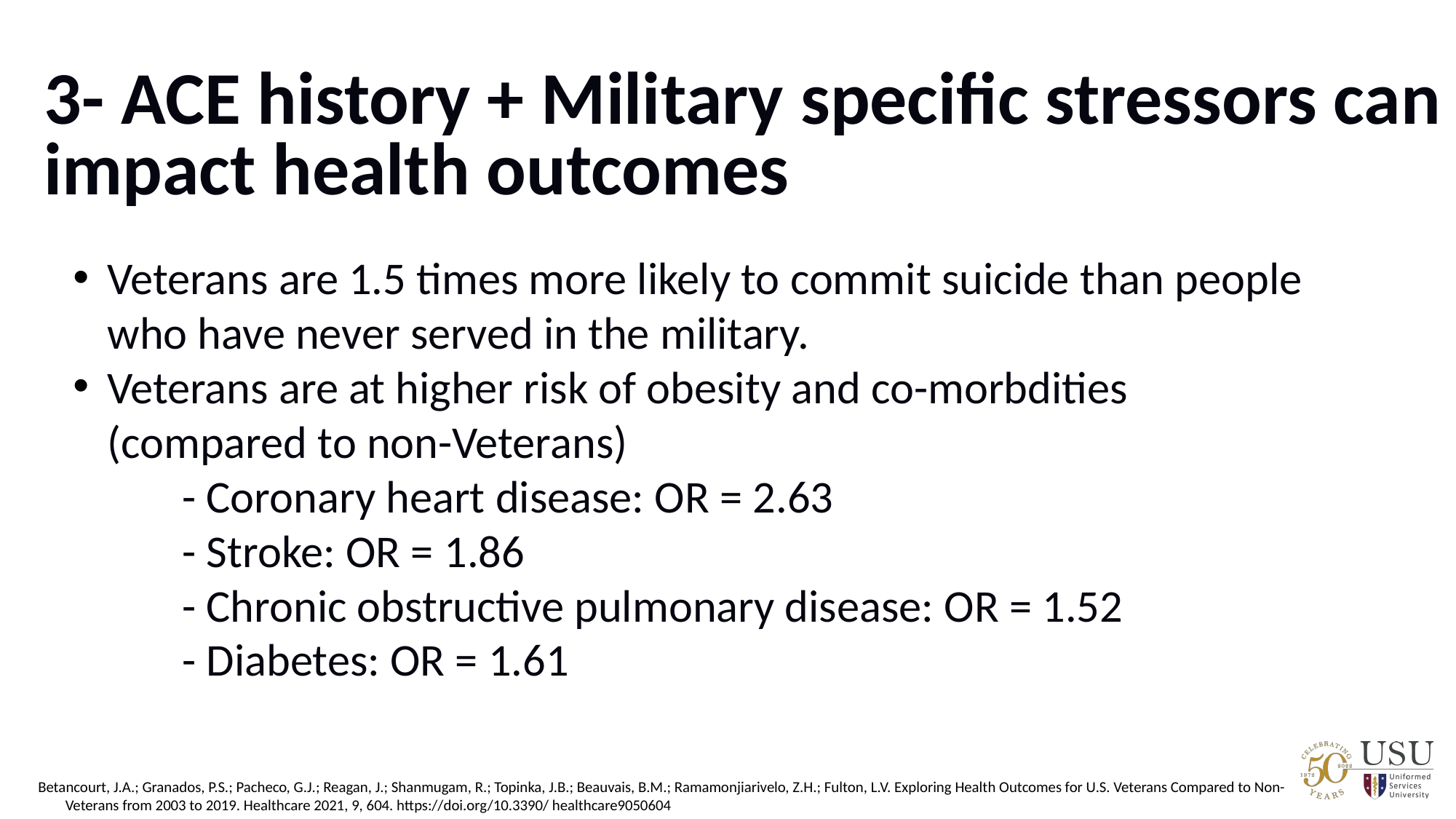

3- ACE history + Military specific stressors can impact health outcomes
Veterans are 1.5 times more likely to commit suicide than people who have never served in the military.
Veterans are at higher risk of obesity and co-morbdities (compared to non-Veterans)
	- Coronary heart disease: OR = 2.63
	- Stroke: OR = 1.86
	- Chronic obstructive pulmonary disease: OR = 1.52
	- Diabetes: OR = 1.61
Betancourt, J.A.; Granados, P.S.; Pacheco, G.J.; Reagan, J.; Shanmugam, R.; Topinka, J.B.; Beauvais, B.M.; Ramamonjiarivelo, Z.H.; Fulton, L.V. Exploring Health Outcomes for U.S. Veterans Compared to Non-Veterans from 2003 to 2019. Healthcare 2021, 9, 604. https://doi.org/10.3390/ healthcare9050604

## Slide 26
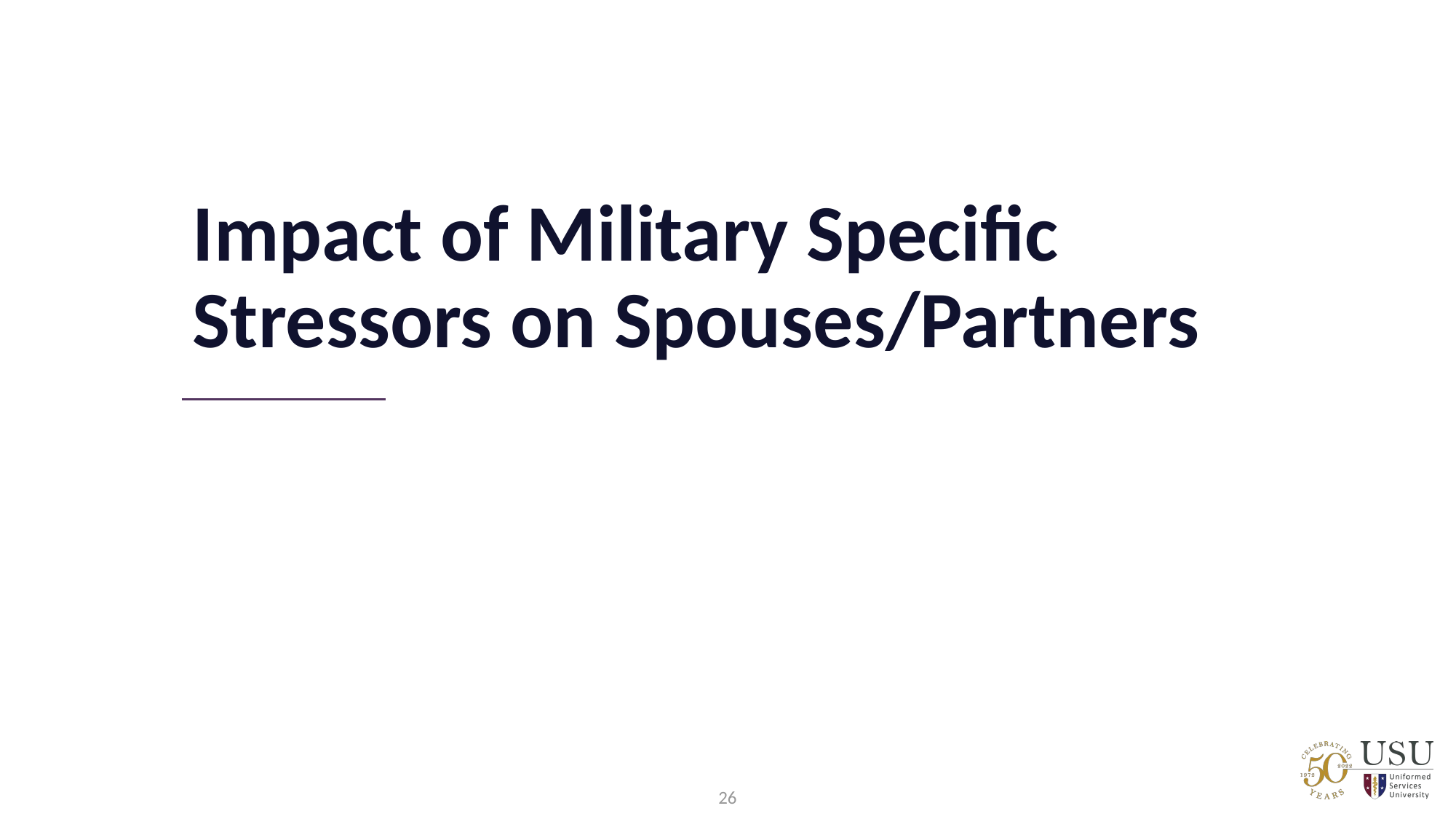

# Impact of Military Specific Stressors on Spouses/Partners
26

## Slide 27
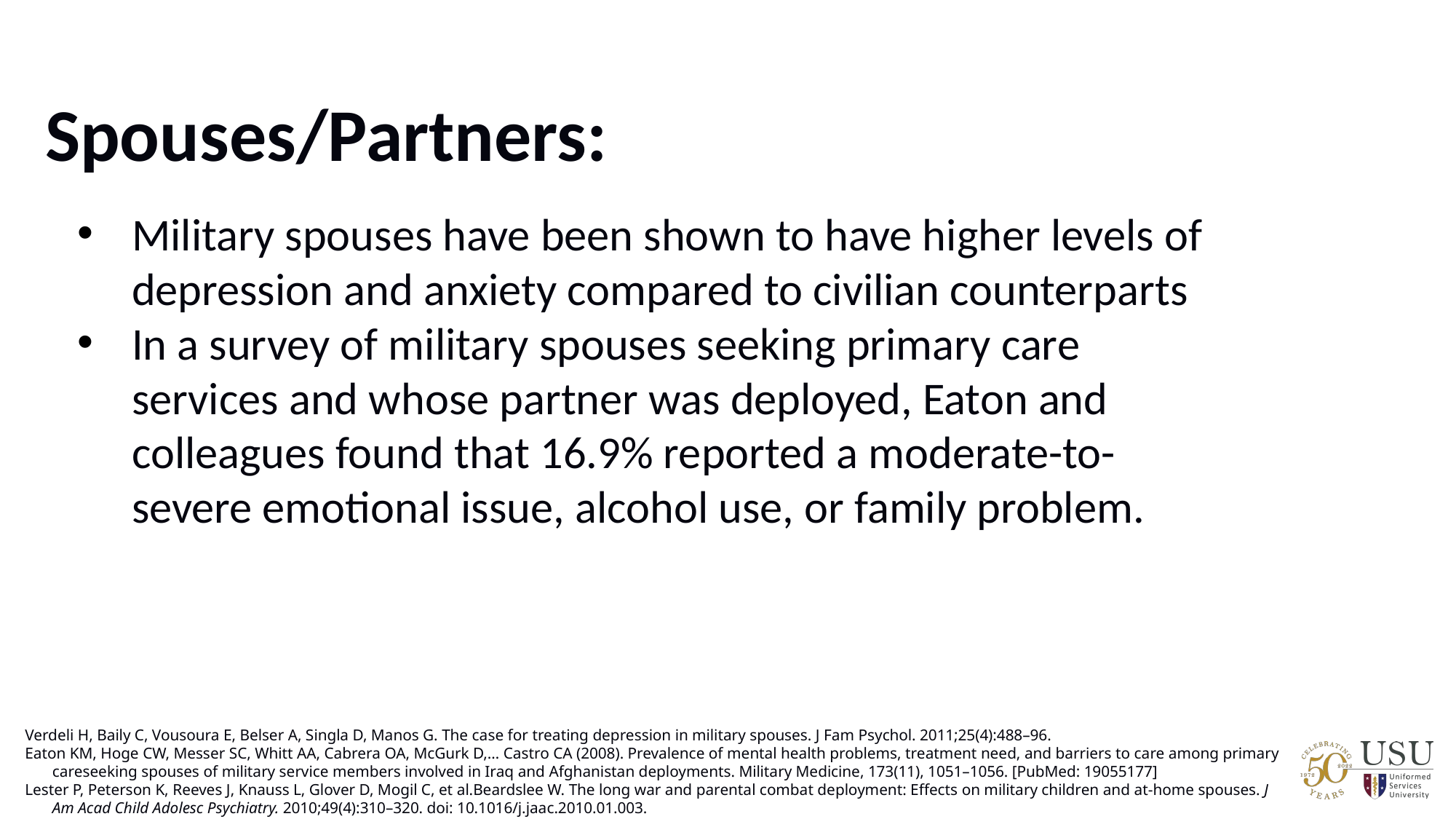

Spouses/Partners:
Military spouses have been shown to have higher levels of depression and anxiety compared to civilian counterparts
In a survey of military spouses seeking primary care services and whose partner was deployed, Eaton and colleagues found that 16.9% reported a moderate-to-severe emotional issue, alcohol use, or family problem.
Verdeli H, Baily C, Vousoura E, Belser A, Singla D, Manos G. The case for treating depression in military spouses. J Fam Psychol. 2011;25(4):488–96.
Eaton KM, Hoge CW, Messer SC, Whitt AA, Cabrera OA, McGurk D,… Castro CA (2008). Prevalence of mental health problems, treatment need, and barriers to care among primary careseeking spouses of military service members involved in Iraq and Afghanistan deployments. Military Medicine, 173(11), 1051–1056. [PubMed: 19055177]
Lester P, Peterson K, Reeves J, Knauss L, Glover D, Mogil C, et al.Beardslee W. The long war and parental combat deployment: Effects on military children and at-home spouses. J Am Acad Child Adolesc Psychiatry. 2010;49(4):310–320. doi: 10.1016/j.jaac.2010.01.003.

## Slide 28
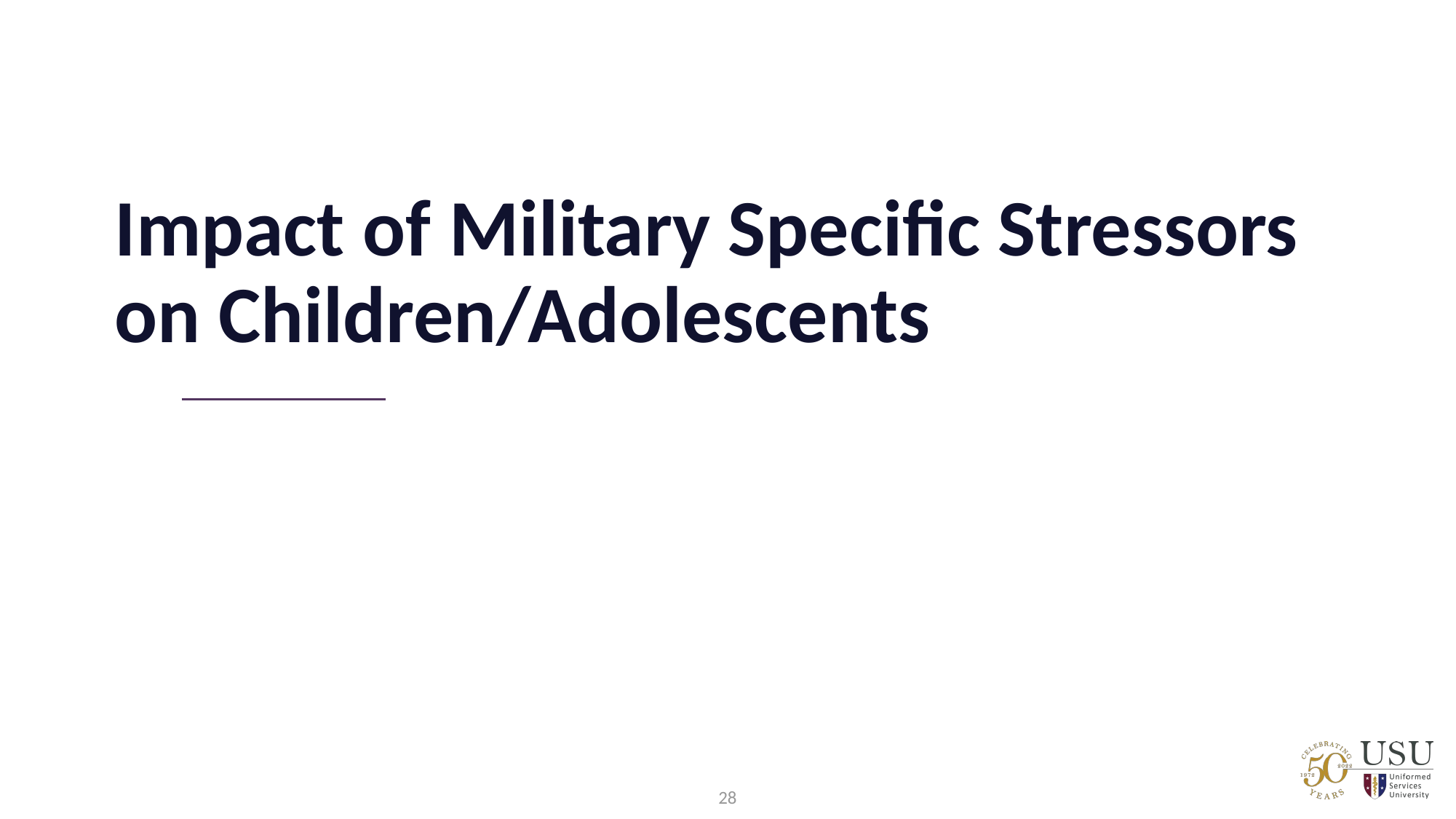

# Impact of Military Specific Stressors on Children/Adolescents
28

## Slide 29
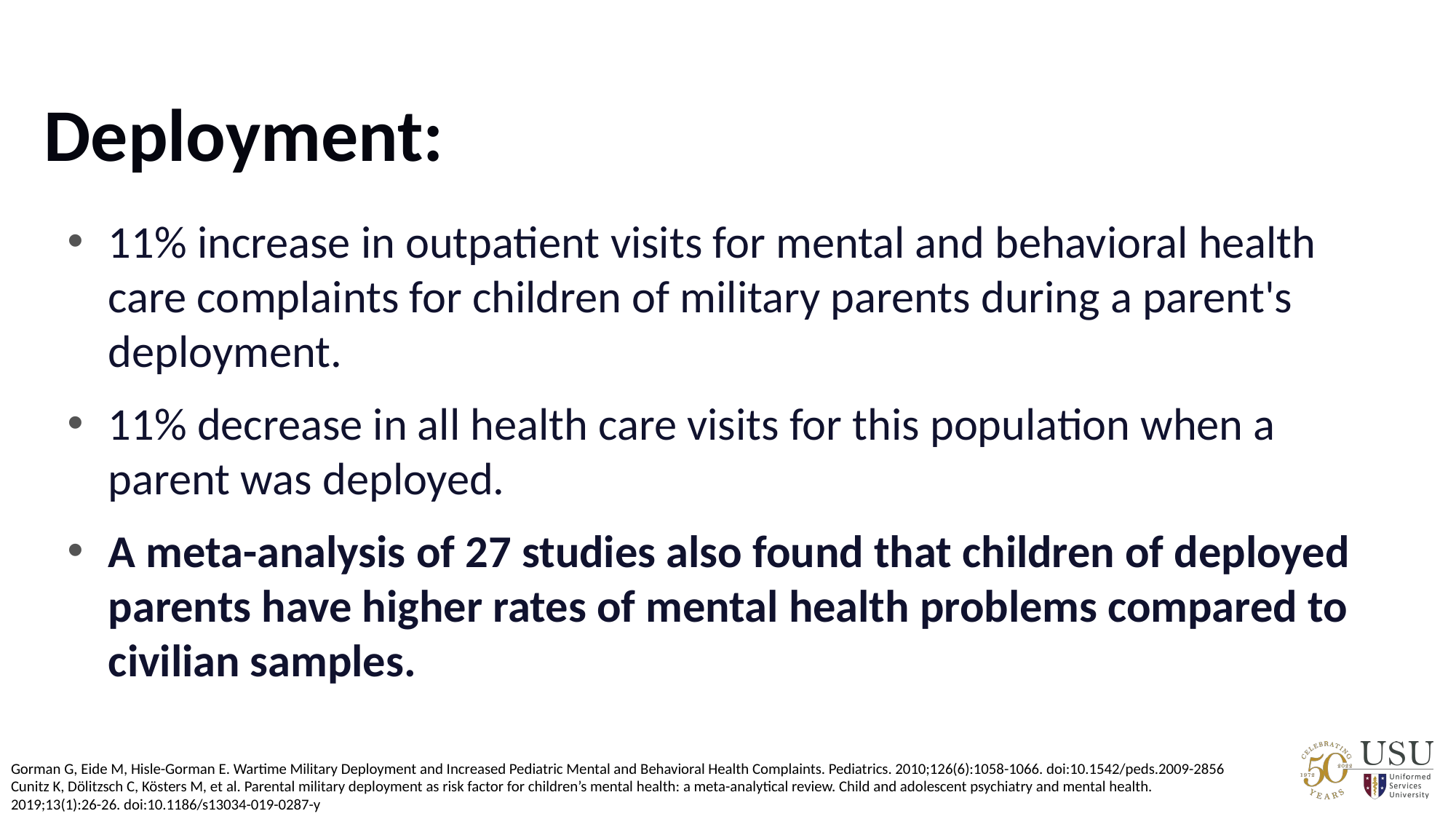

Deployment:
11% increase in outpatient visits for mental and behavioral health care complaints for children of military parents during a parent's deployment.
11% decrease in all health care visits for this population when a parent was deployed.
A meta-analysis of 27 studies also found that children of deployed parents have higher rates of mental health problems compared to civilian samples.
Gorman G, Eide M, Hisle-Gorman E. Wartime Military Deployment and Increased Pediatric Mental and Behavioral Health Complaints. Pediatrics. 2010;126(6):1058-1066. doi:10.1542/peds.2009-2856
Cunitz K, Dölitzsch C, Kösters M, et al. Parental military deployment as risk factor for children’s mental health: a meta-analytical review. Child and adolescent psychiatry and mental health. 2019;13(1):26-26. doi:10.1186/s13034-019-0287-y

## Slide 30
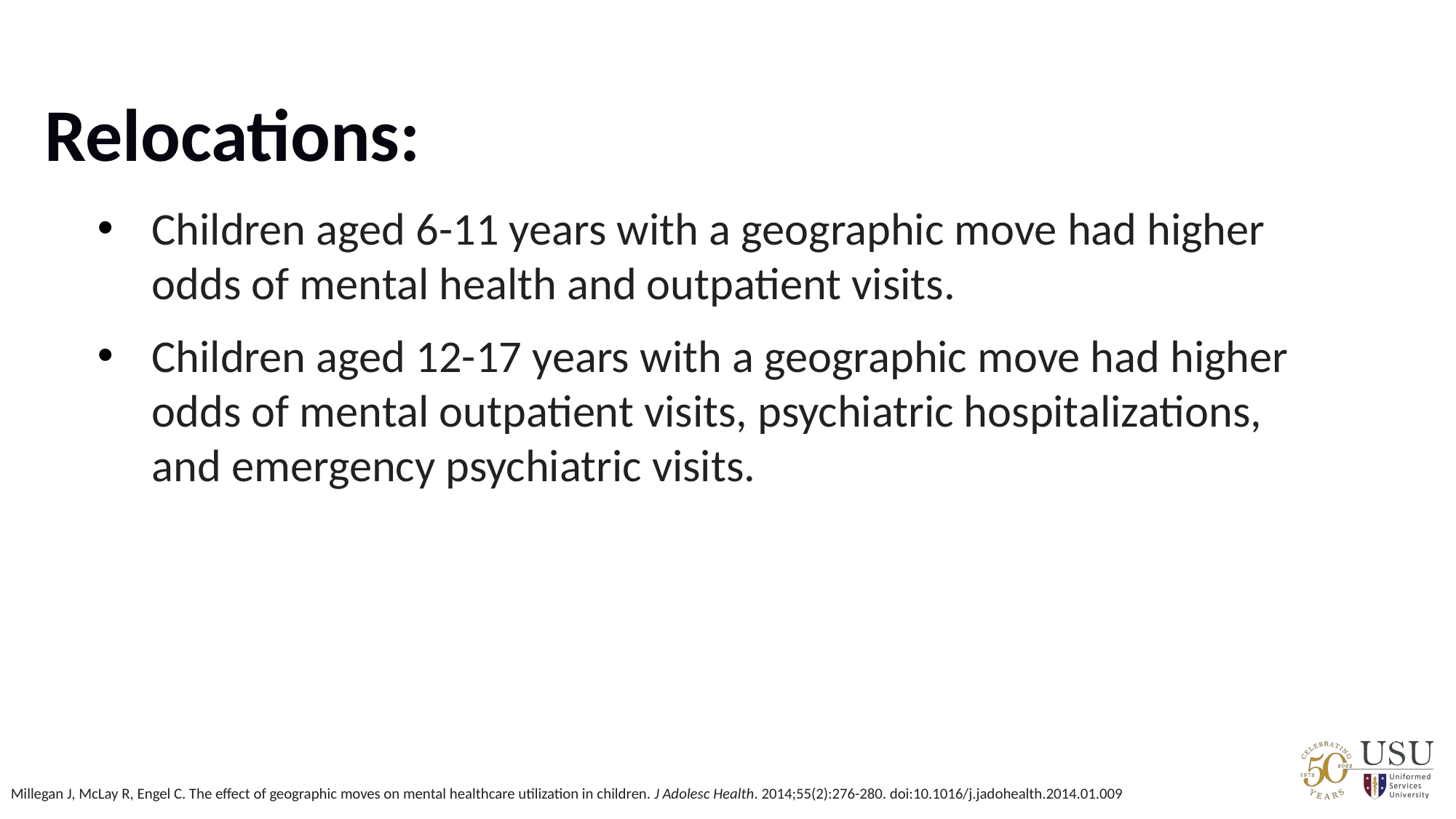

Relocations:
Children aged 6-11 years with a geographic move had higher odds of mental health and outpatient visits.
Children aged 12-17 years with a geographic move had higher odds of mental outpatient visits, psychiatric hospitalizations, and emergency psychiatric visits.
Millegan J, McLay R, Engel C. The effect of geographic moves on mental healthcare utilization in children. J Adolesc Health. 2014;55(2):276-280. doi:10.1016/j.jadohealth.2014.01.009

## Slide 31
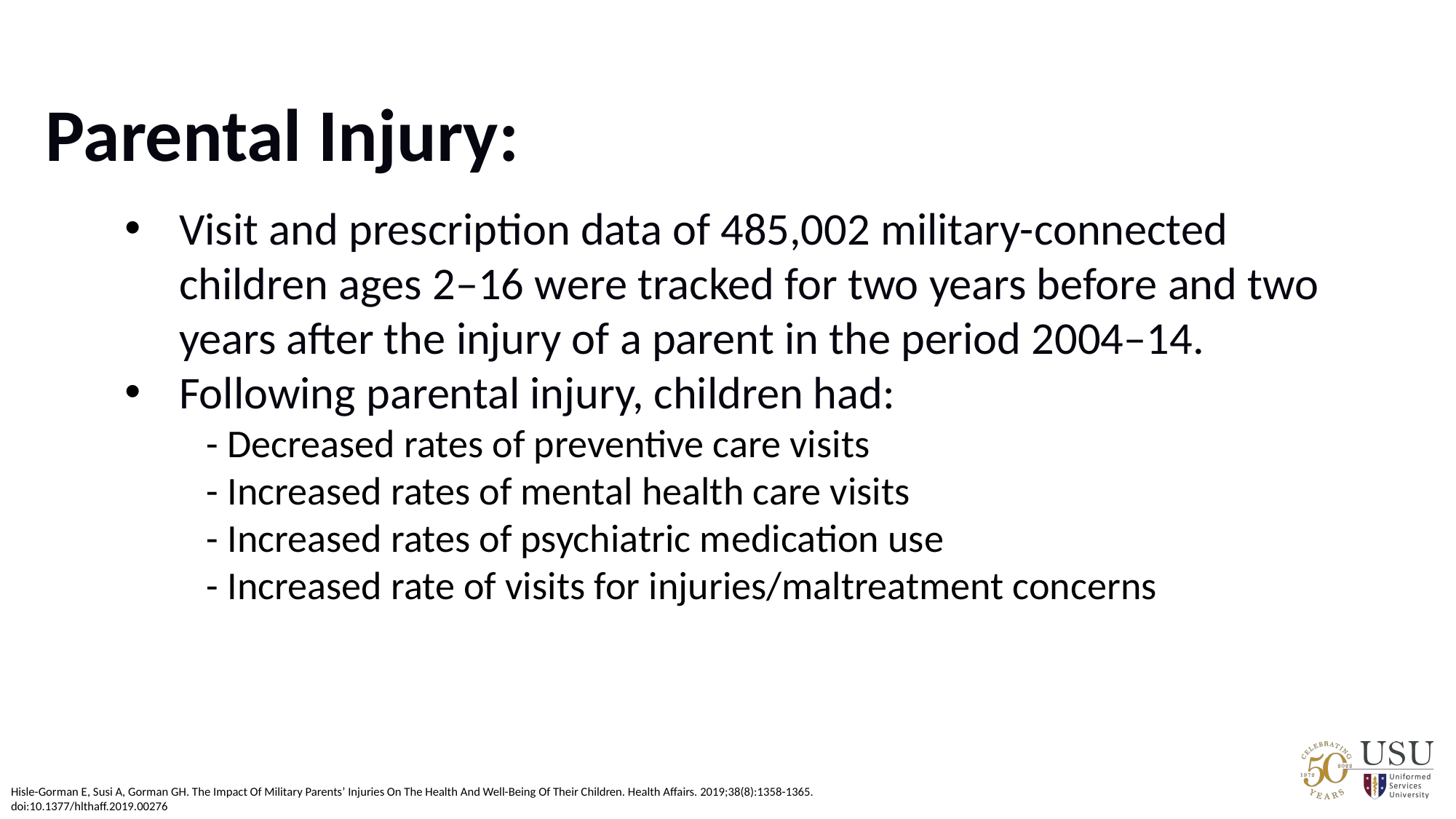

Parental Injury:
Visit and prescription data of 485,002 military-connected children ages 2–16 were tracked for two years before and two years after the injury of a parent in the period 2004–14.
Following parental injury, children had:
	- Decreased rates of preventive care visits
	- Increased rates of mental health care visits
	- Increased rates of psychiatric medication use
	- Increased rate of visits for injuries/maltreatment concerns
Hisle-Gorman E, Susi A, Gorman GH. The Impact Of Military Parents’ Injuries On The Health And Well-Being Of Their Children. Health Affairs. 2019;38(8):1358-1365. doi:10.1377/hlthaff.2019.00276

## Slide 32
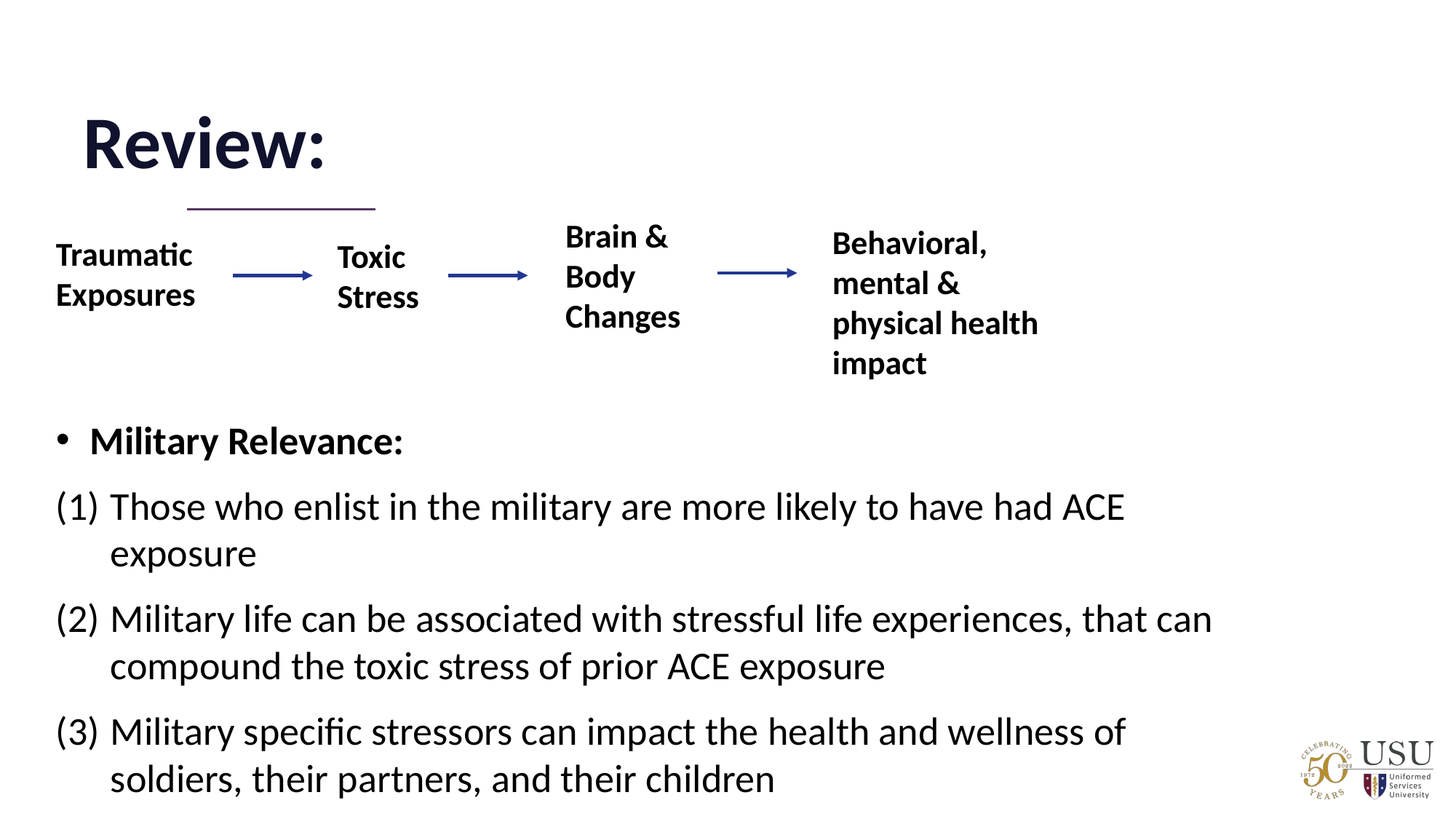

# Review:
Brain &
Body
Changes
Behavioral, mental & physical health impact
Traumatic Exposures
Toxic
Stress
Military Relevance:
Those who enlist in the military are more likely to have had ACE exposure
Military life can be associated with stressful life experiences, that can compound the toxic stress of prior ACE exposure
Military specific stressors can impact the health and wellness of soldiers, their partners, and their children

## Slide 33
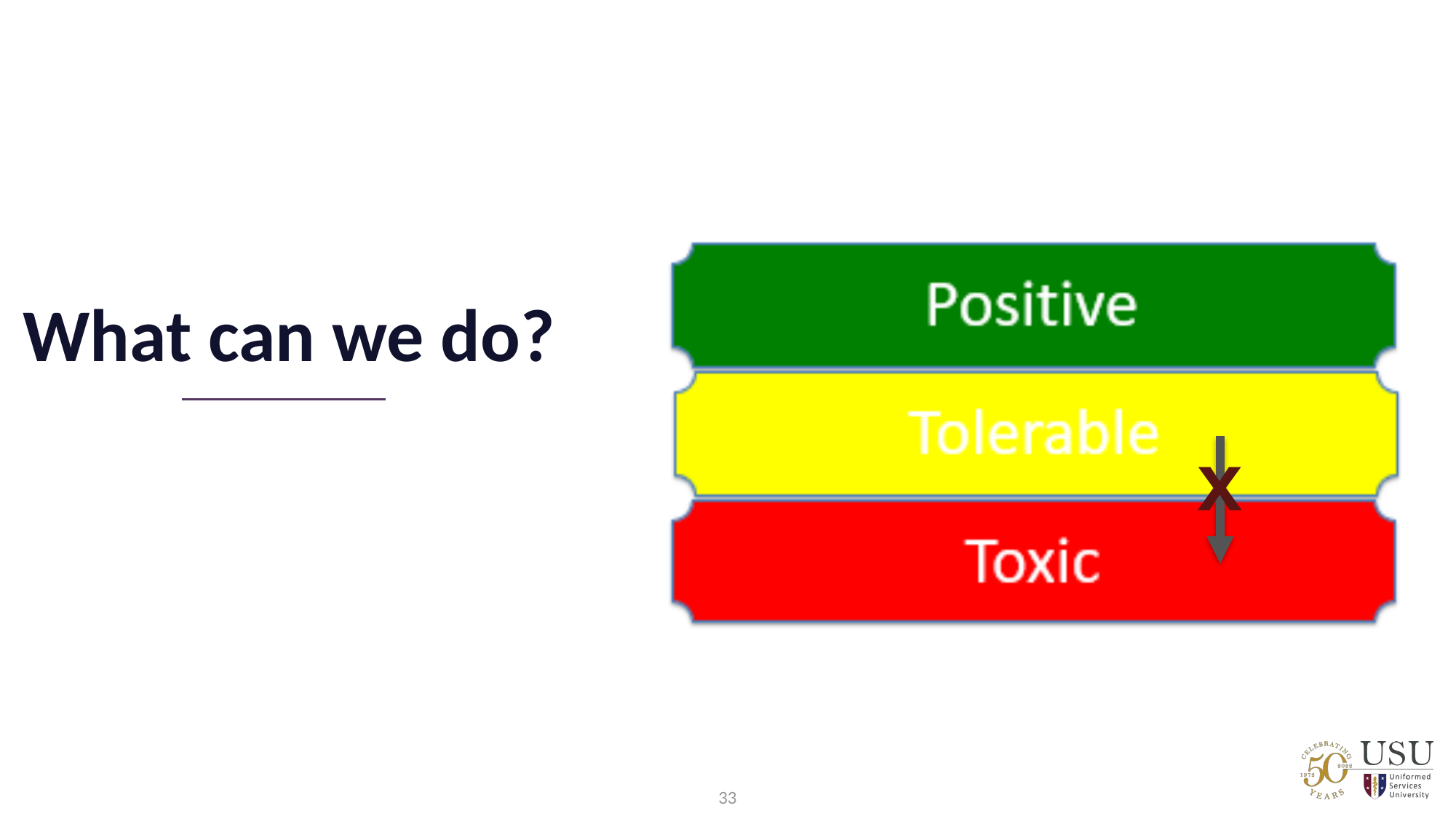

# What can we do?
x
x
33

## Slide 34
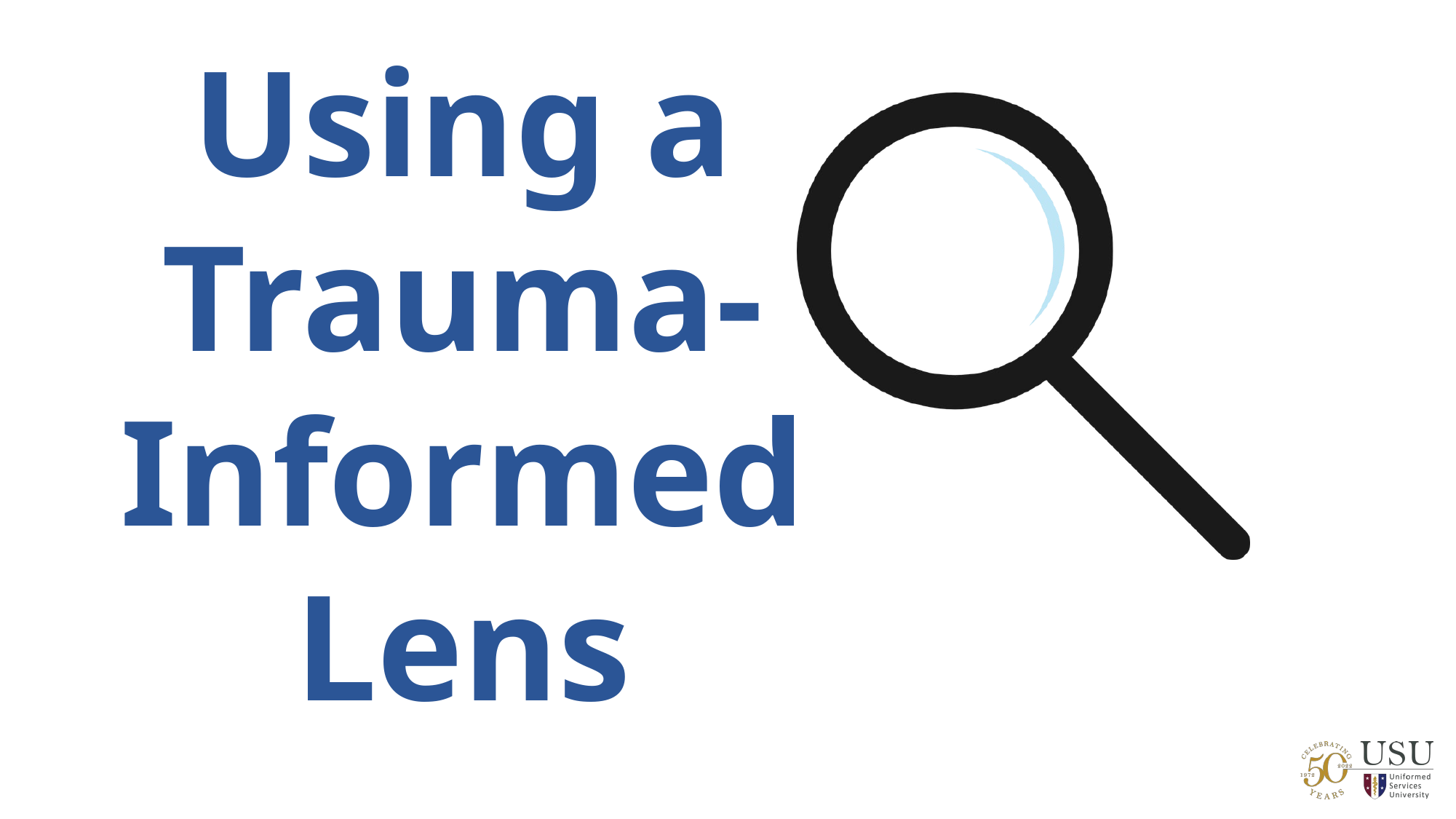

Using a Trauma-Informed Lens

## Slide 35
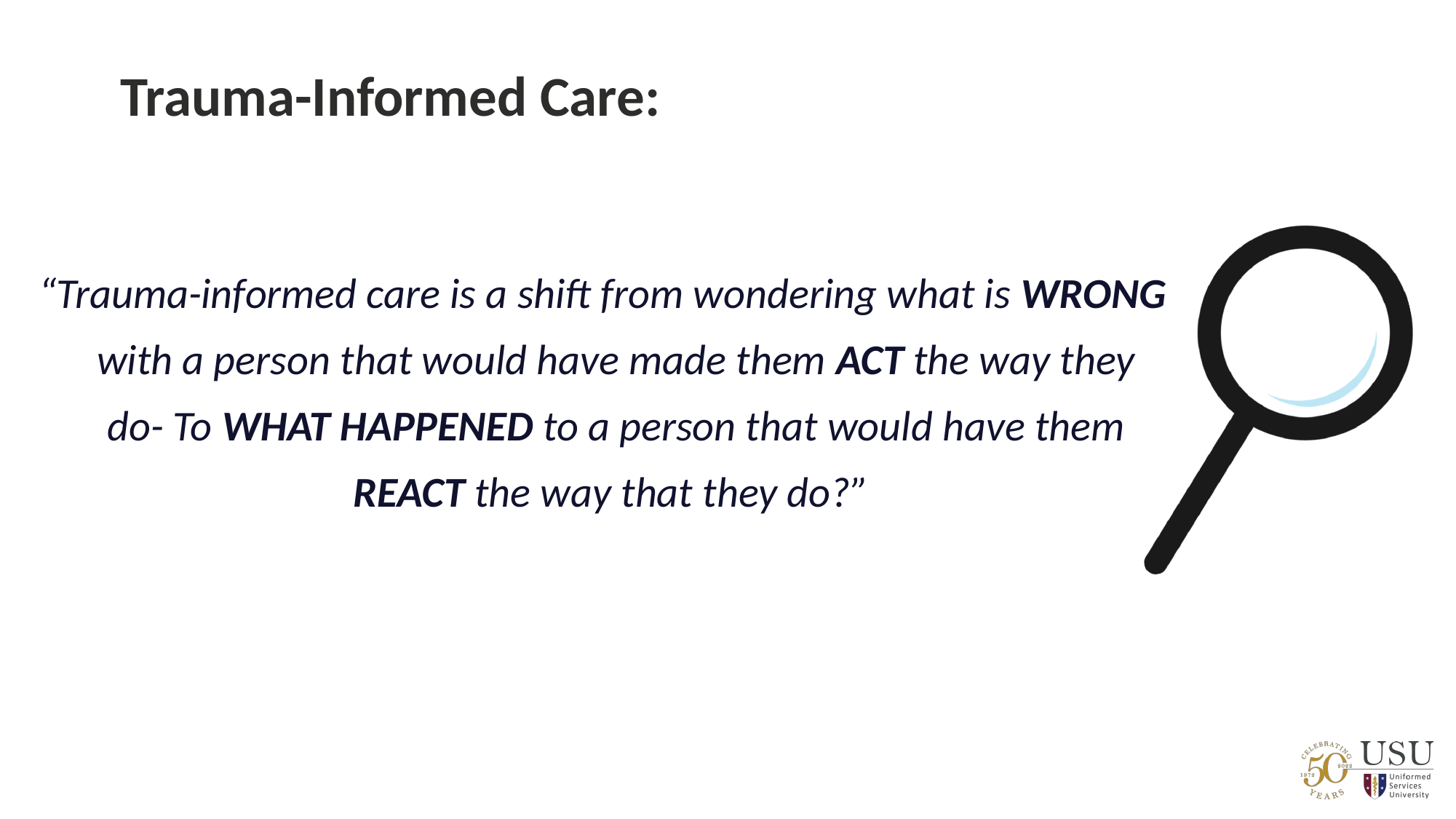

Trauma-Informed Care:
“Trauma-informed care is a shift from wondering what is WRONG with a person that would have made them ACT the way they do- To WHAT HAPPENED to a person that would have them REACT the way that they do?”

## Slide 36
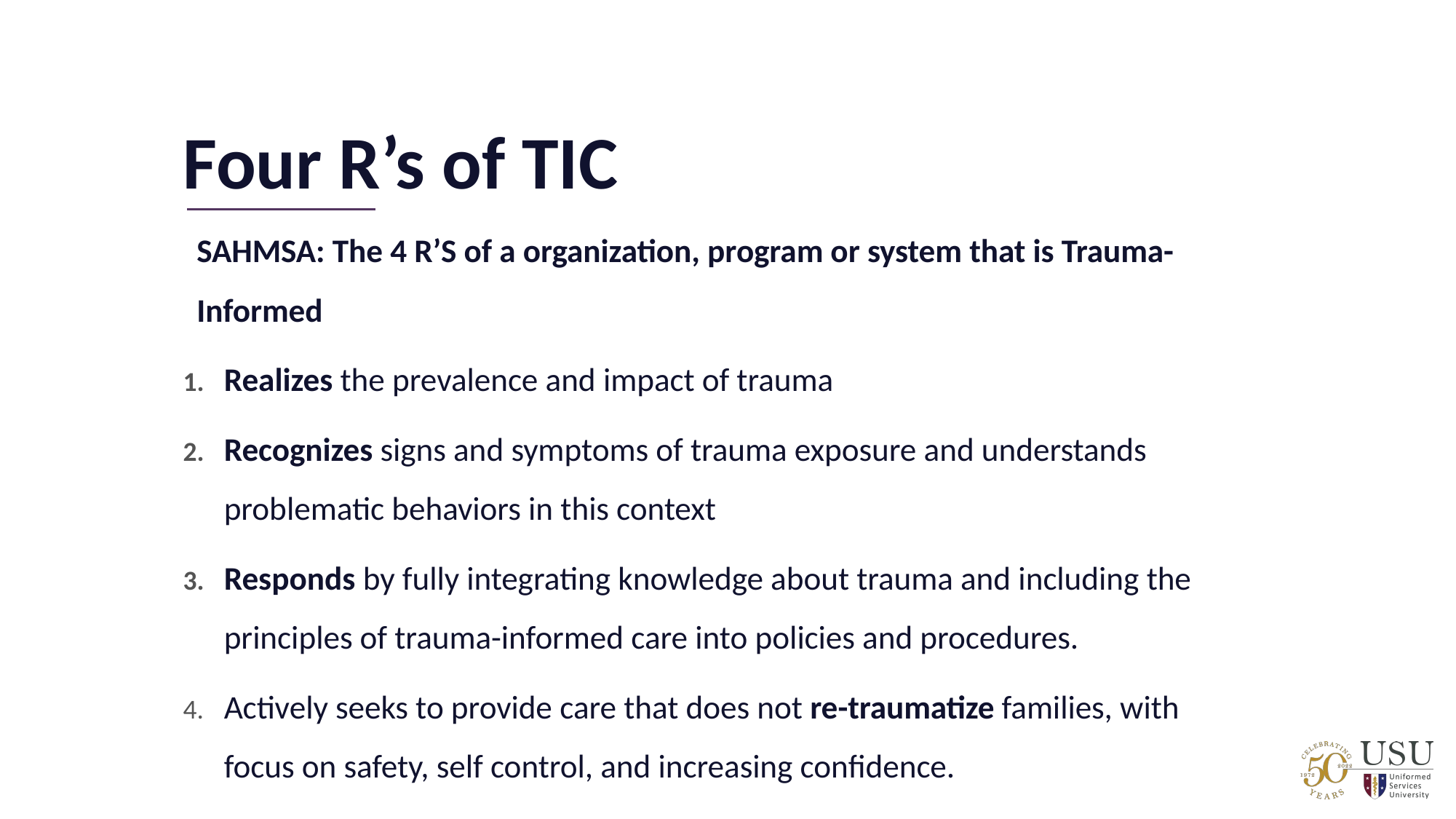

# Four R’s of TIC
SAHMSA: The 4 R’S of a organization, program or system that is Trauma-Informed
Realizes the prevalence and impact of trauma
Recognizes signs and symptoms of trauma exposure and understands problematic behaviors in this context
Responds by fully integrating knowledge about trauma and including the principles of trauma-informed care into policies and procedures.
Actively seeks to provide care that does not re-traumatize families, with focus on safety, self control, and increasing confidence.

## Slide 37
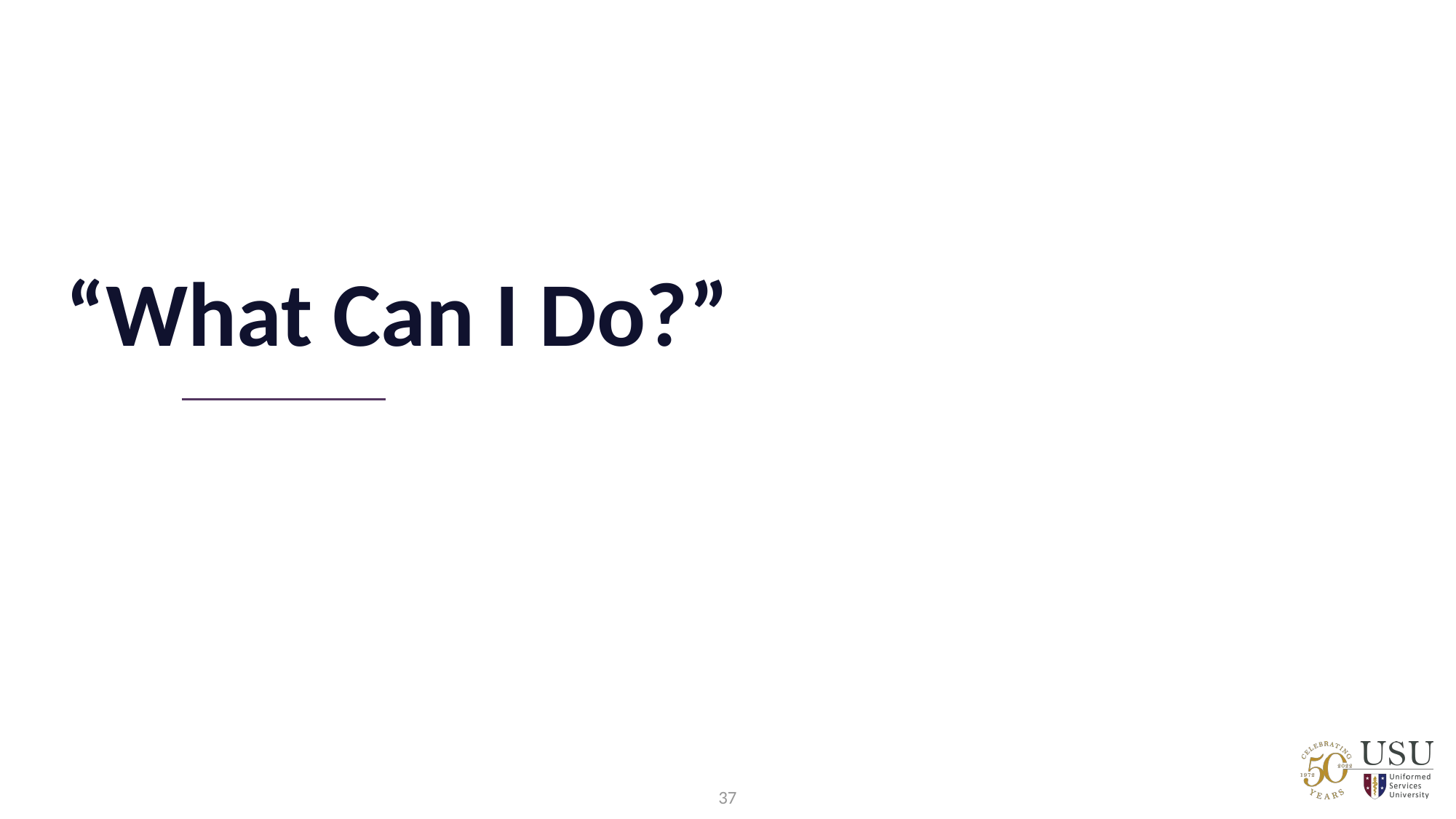

# “What Can I Do?”
37

## Slide 38
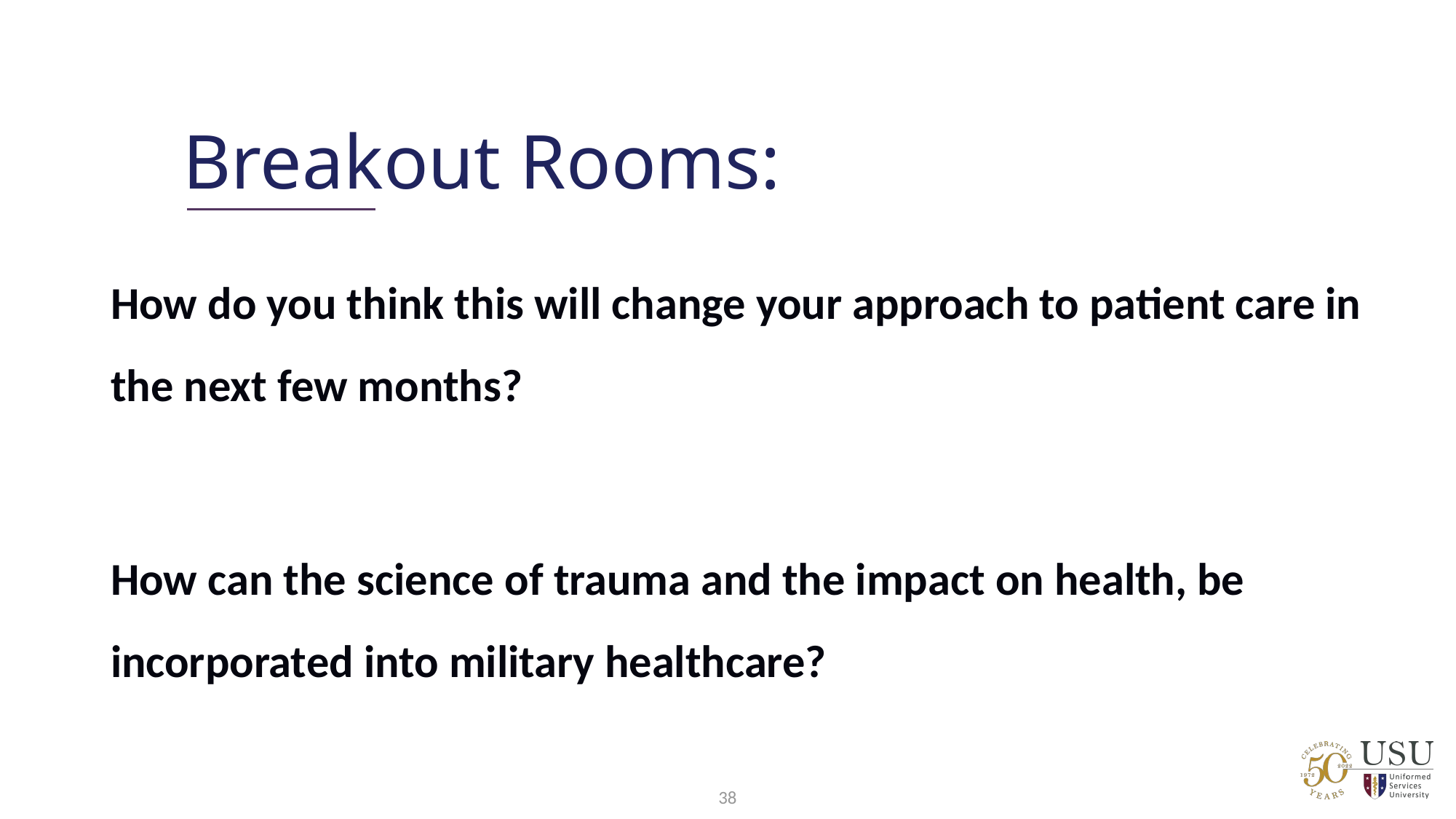

# Breakout Rooms:
How do you think this will change your approach to patient care in the next few months?
How can the science of trauma and the impact on health, be incorporated into military healthcare?
38

## Slide 39
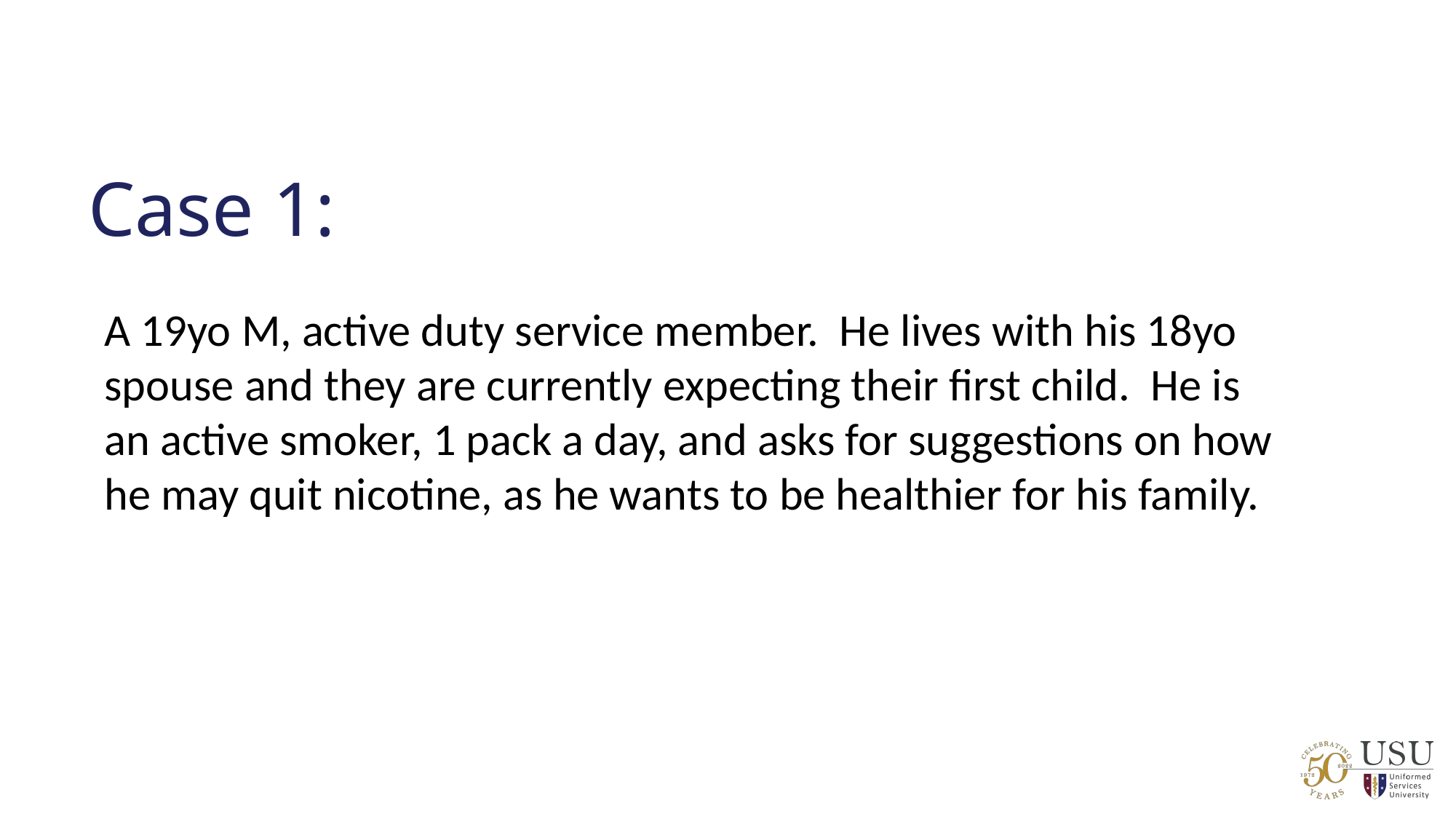

# Case 1:
A 19yo M, active duty service member. He lives with his 18yo spouse and they are currently expecting their first child. He is an active smoker, 1 pack a day, and asks for suggestions on how he may quit nicotine, as he wants to be healthier for his family.

## Slide 40
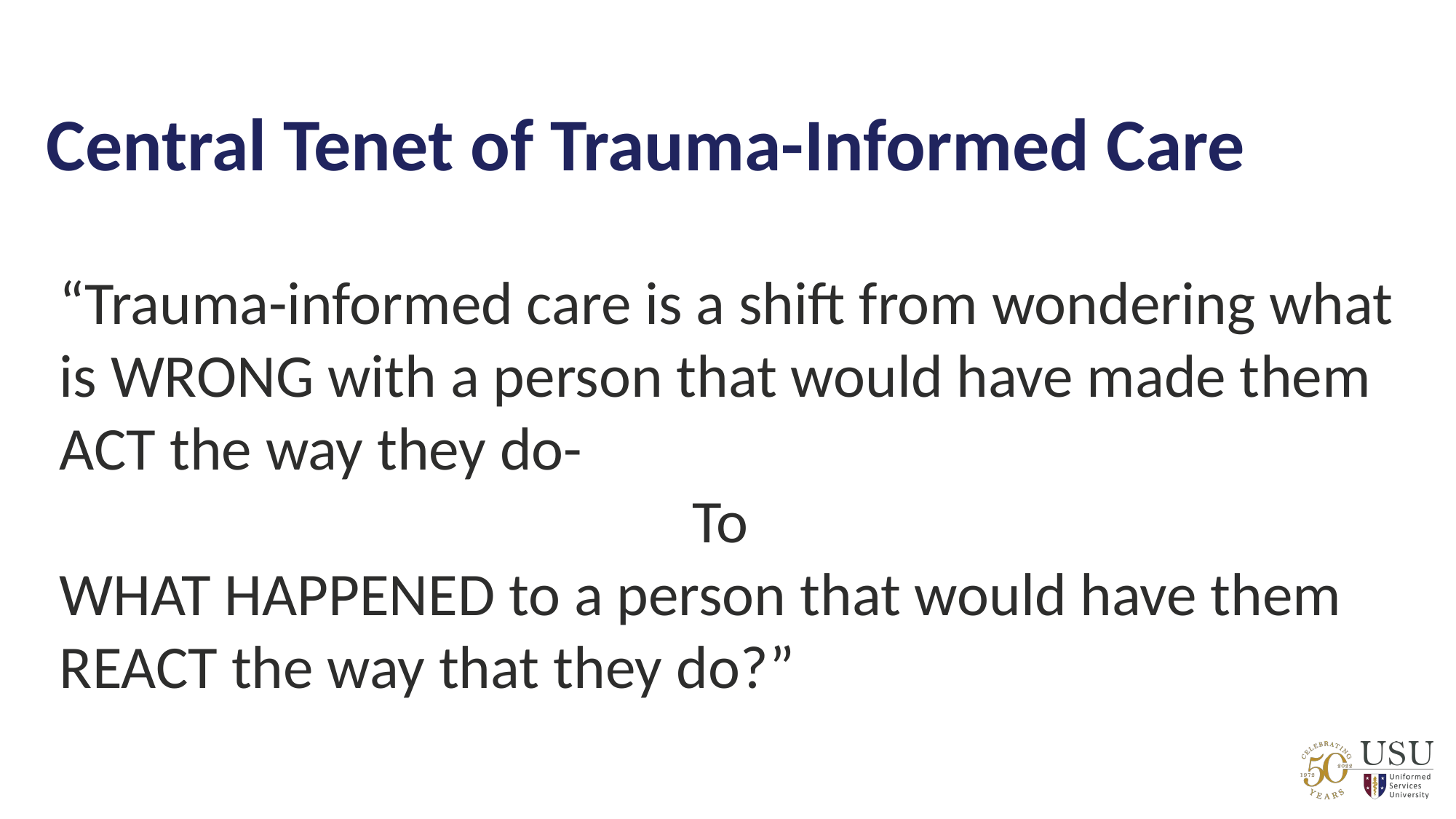

# Central Tenet of Trauma-Informed Care
“Trauma-informed care is a shift from wondering what is WRONG with a person that would have made them ACT the way they do-
To
WHAT HAPPENED to a person that would have them REACT the way that they do?”

## Slide 41
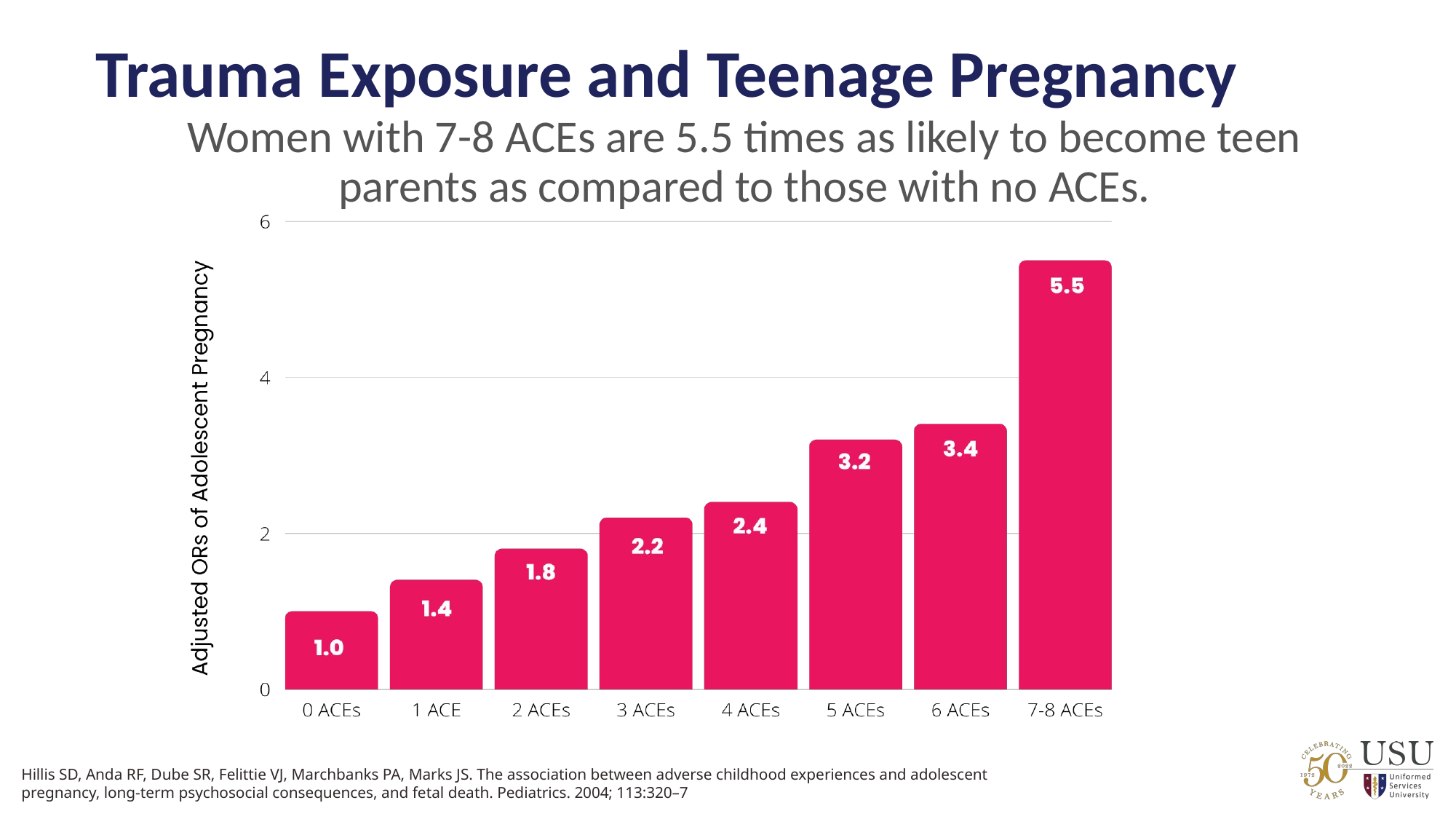

Trauma Exposure and Teenage Pregnancy
Women with 7-8 ACEs are 5.5 times as likely to become teen parents as compared to those with no ACEs.
Hillis SD, Anda RF, Dube SR, Felittie VJ, Marchbanks PA, Marks JS. The association between adverse childhood experiences and adolescent pregnancy, long-term psychosocial consequences, and fetal death. Pediatrics. 2004; 113:320–7

## Slide 42
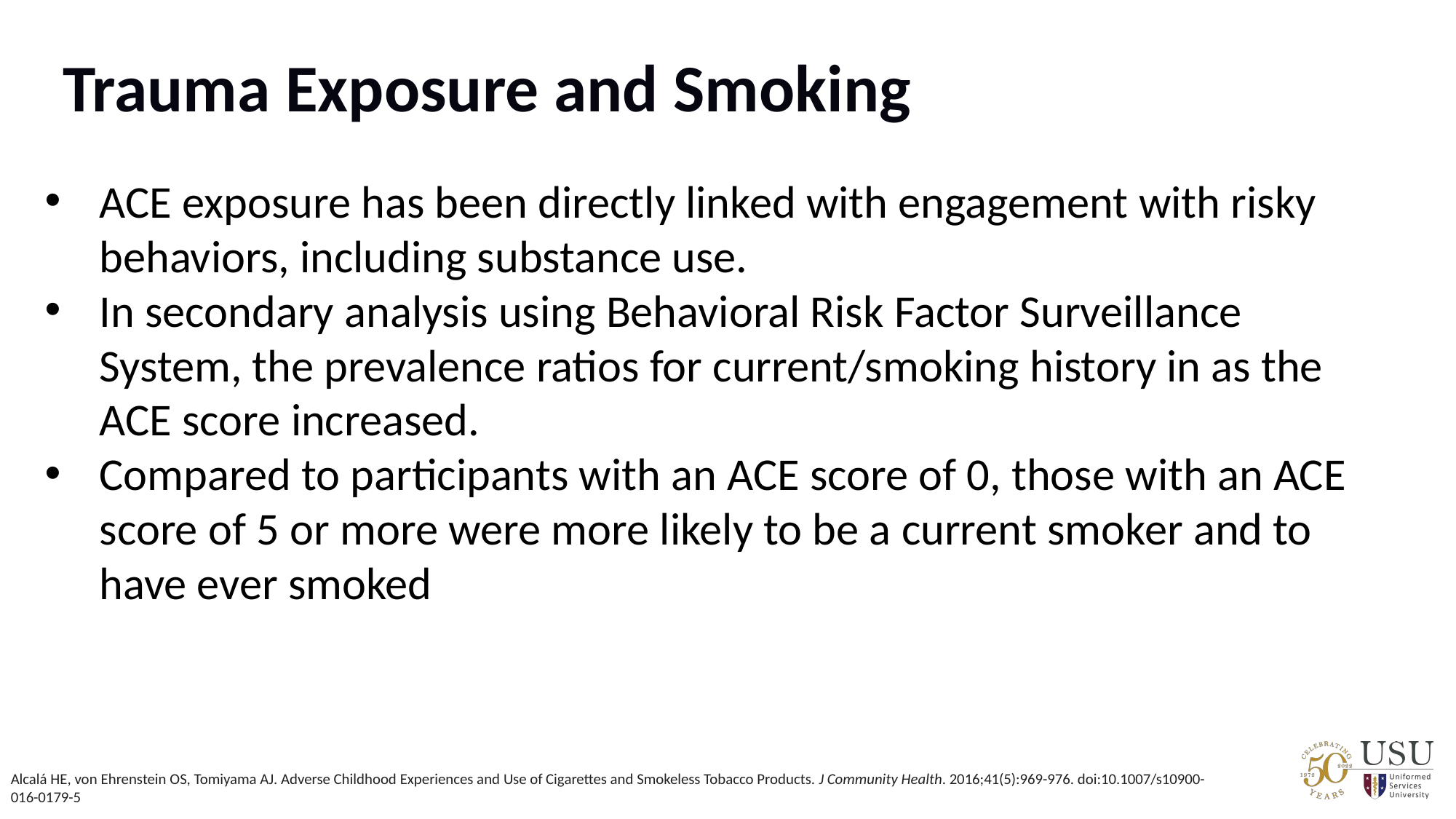

Trauma Exposure and Smoking
ACE exposure has been directly linked with engagement with risky behaviors, including substance use.
In secondary analysis using Behavioral Risk Factor Surveillance System, the prevalence ratios for current/smoking history in as the ACE score increased.
Compared to participants with an ACE score of 0, those with an ACE score of 5 or more were more likely to be a current smoker and to have ever smoked
Alcalá HE, von Ehrenstein OS, Tomiyama AJ. Adverse Childhood Experiences and Use of Cigarettes and Smokeless Tobacco Products. J Community Health. 2016;41(5):969-976. doi:10.1007/s10900-016-0179-5

## Slide 43
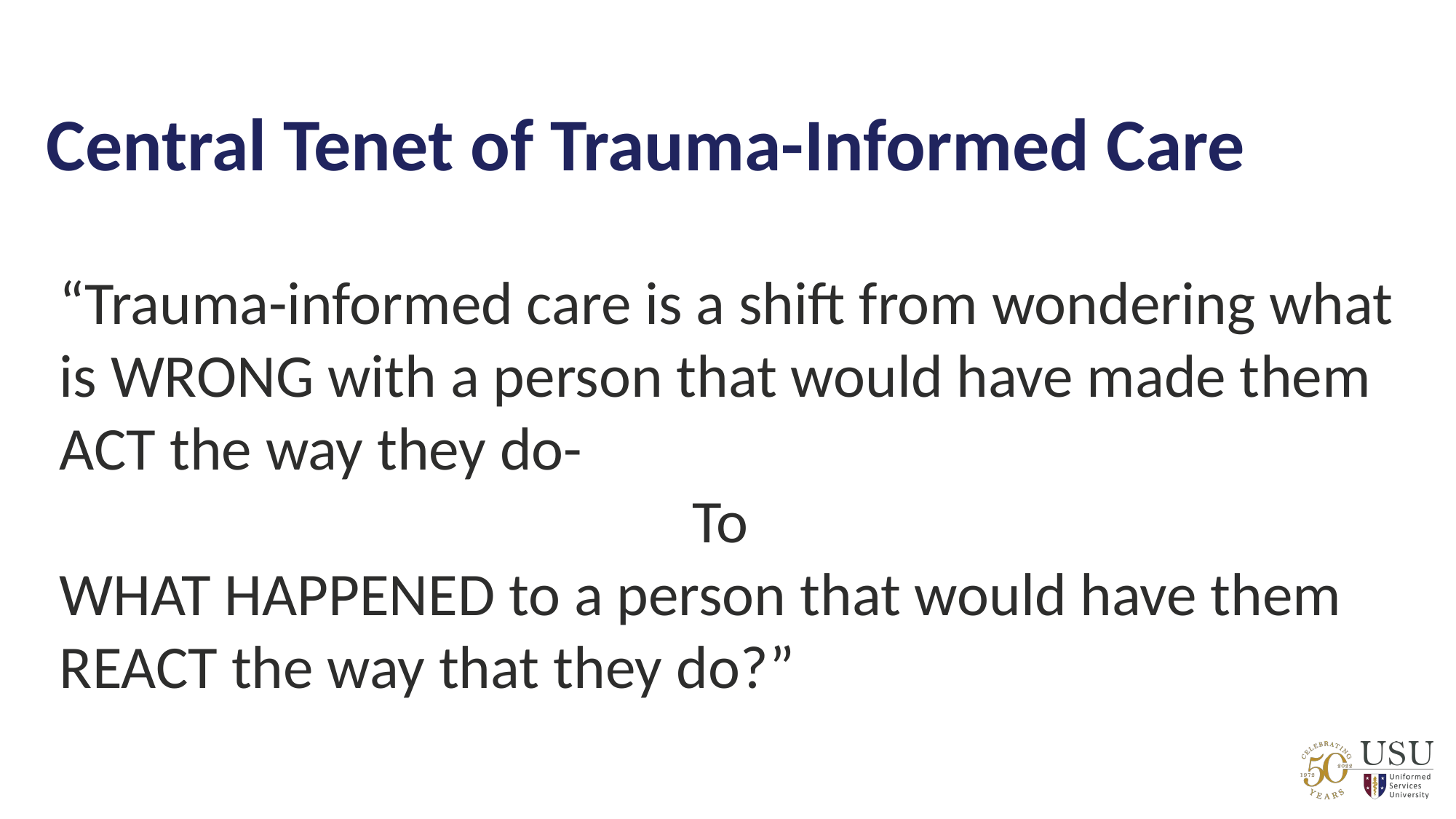

# Central Tenet of Trauma-Informed Care
“Trauma-informed care is a shift from wondering what is WRONG with a person that would have made them ACT the way they do-
To
WHAT HAPPENED to a person that would have them REACT the way that they do?”

## Slide 44
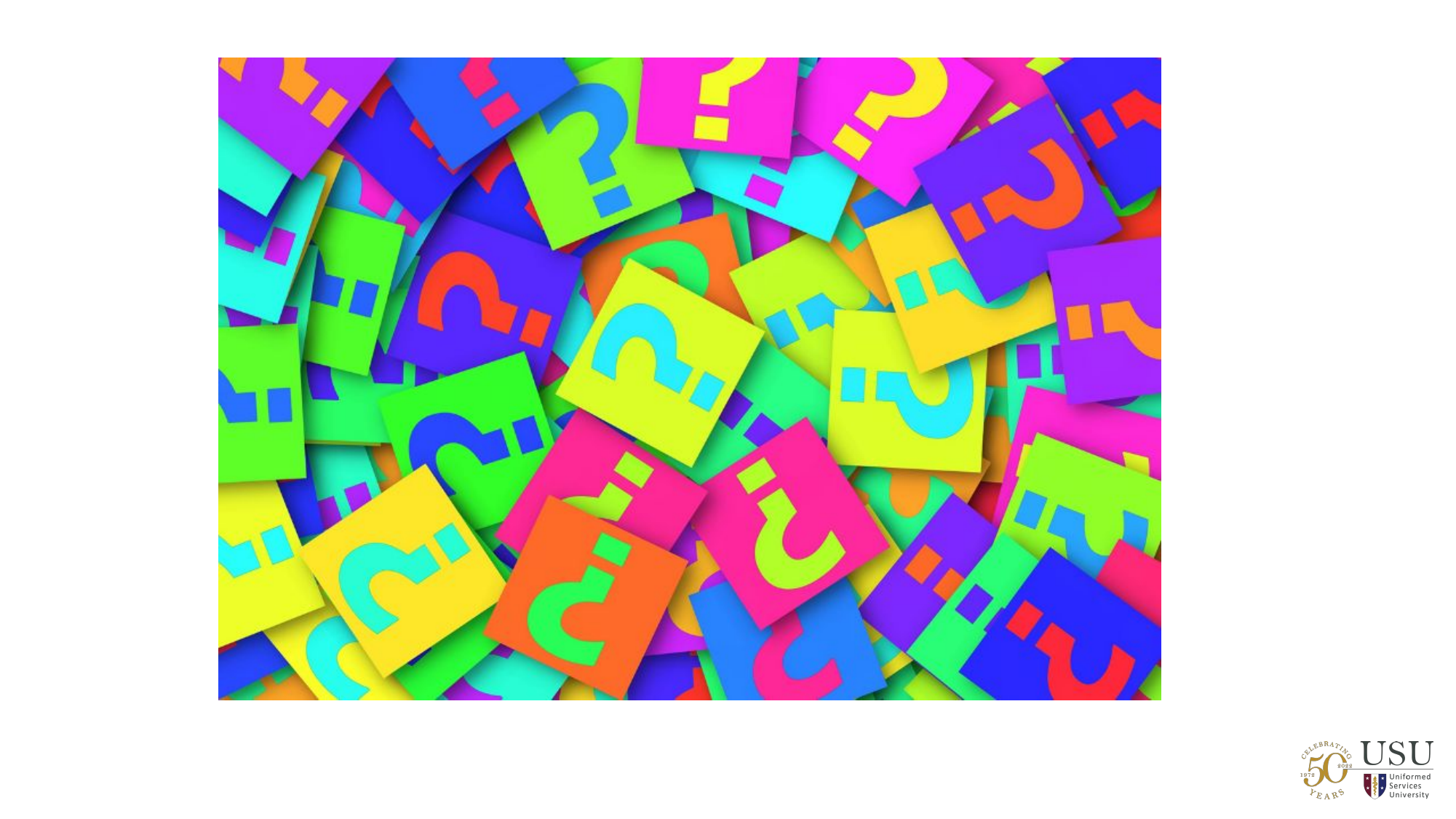

Supplement: Supplementary file 1 — Slide Set.pptxPresession Message.docxPre-Post Evaluation.docxFacilitator Guide.docx [file mep_2374-8265.11466-s001.zip › A. Slide Set.pptx]
